# Baseline microbiota composition modulates antibiotic-mediated effects on the gut microbiota and host

**DOI:** 10.1186/s40168-019-0725-3

**Published:** 2019-08-02

**Authors:** Aonghus Lavelle, Thomas Walter Hoffmann, Hang-Phuong Pham, Philippe Langella, Eric Guédon, Harry Sokol

**Affiliations:** 1 Sorbonne Université, INSERM, Saint-Antoine Research Center (CRSA), Paris, France; 2 INRA, UMR1319 Micalis, AgroParisTech, Jouy-en-Josas, France; 3 ILTOO Pharma, 14 rue des reculettes, Paris, France; 4 STLO, INRA, Agrocampus Ouest, Rennes, France; 5 Department of Gastroenterology, Saint Antoine Hospital, Assistance Publique – Hopitaux de Paris, Sorbonne Universités, 184 rue du Faubourg Saint-Antoine, 75571 Paris CEDEX 12, Paris, France

**Keywords:** Microbiome, Antibiotics, Transcriptome, Gut

## Abstract

**Background:**

Normal mammalian development and homeostasis are dependent upon the gut microbiota. Antibiotics, essential for the treatment and prophylaxis of bacterial infections, can have collateral effects on the gut microbiota composition, which can in turn have far-reaching and potentially deleterious consequences for the host. However, the magnitude and duration of such collateral effects appear to vary between individuals. Furthermore, the degree to which such perturbations affect the host response is currently unclear. We aimed to test the hypothesis that different human microbiomes have different responses to a commonly prescribed antibiotic and that these differences may impact the host response.

**Methods:**

Germ-free mice (*n* = 30) humanized with the microbiota of two unrelated donors (A and B) were subjected to a 7-day antibiotic challenge with amoxicillin-clavulanate (“co-amoxiclav”). Microbiome and colonic transcriptome analysis was performed, pre (day 0) and post antibiotics (day 8) and subsequently into recovery (days 11 and 18).

**Results:**

Unique community profiles were evident depending upon the donor, with donor A recipient mice being dominated by *Prevotella* and *Faecalibacterium* and donor B recipient mice dominated by *Bacteroides* and *Parabacteroides*. Donor A mice underwent a marked destabilization of their microbiota following antibiotic treatment, while donor B mice maintained a more stable profile. Dramatic and overlapping alterations in the host transcriptome were apparent following antibiotic challenge in both groups. Despite this overlap, donor A mice experienced a more significant alteration in gene expression and uniquely showed correlations between host pathways and key microbial genera.

**Conclusions:**

Germ-free mice humanized by different donor microbiotas maintain distinct microbiome profiles, which respond in distinct ways to antibiotic challenge and evince host responses that parallel microbiome disequilibrium. These results suggest that inter-individual variation in the gut microbiota may contribute to personalized host responses following microbiota perturbation.

**Electronic supplementary material:**

The online version of this article (10.1186/s40168-019-0725-3) contains supplementary material, which is available to authorized users.

## Background

The mammalian microbiome incorporates a vast and diverse range of microorganisms that have co-evolved to live with us, contributing essential functions through their collective genetic and metabolic repertoire [1]. Ecological diversity and inter-individual variation are hallmarks of the healthy gut microbiome [2] and appropriate assembly of microbial communities in infancy and their maintenance in adulthood is of critical importance for metabolic [3] and immune [4] maturation, with the microbiome playing a role in both immune education and homeostasis. Many common environmental exposures can have demonstrable effects on the microbiome, including mode of birth delivery [5], antibiotic use [6, 7], reduced dietary diversity [8], and prescription medications [9], and some associations with these exposures and subsequent development of immune, inflammatory, or metabolic conditions have been made [7, 8, 10–13].

Antibiotic treatment, one of the fundamental medical achievements of the past century, represents a notable example of microbiome perturbation, with rapid and sometimes enduring changes to the community structure [14]. Antibiotic administration during the neonatal “window period”—when the gut microbiota is in part responsible for immune education [15]—may have long-term consequences, and early life antibiotics have been associated with increased risk of asthma, obesity, and Crohn’s disease [6, 7, 11, 16]. Furthermore, antibiotic use is directly associated with the development of vancomycin-resistant *Enterococcus* (VRE)[17], recurrent *Clostridium difficile* infection (CDI)[18], and the emergence of antibiotic resistance as a global health threat [19]. Notably, response and recovery of human gut microbiota communities following antibiotic treatment can be highly variable between individuals, as described in the context of responses to the antibiotic ciprofloxacin [20] and the routine prescription of antibiotics clarithromycin and metronidazole for the treatment of *Helicobacter pylori* infection [21]. Whether such changes are restricted to the microbiota or result in a downstream alteration in intestinal host response as well is not currently clear.

**Fig. 1:**
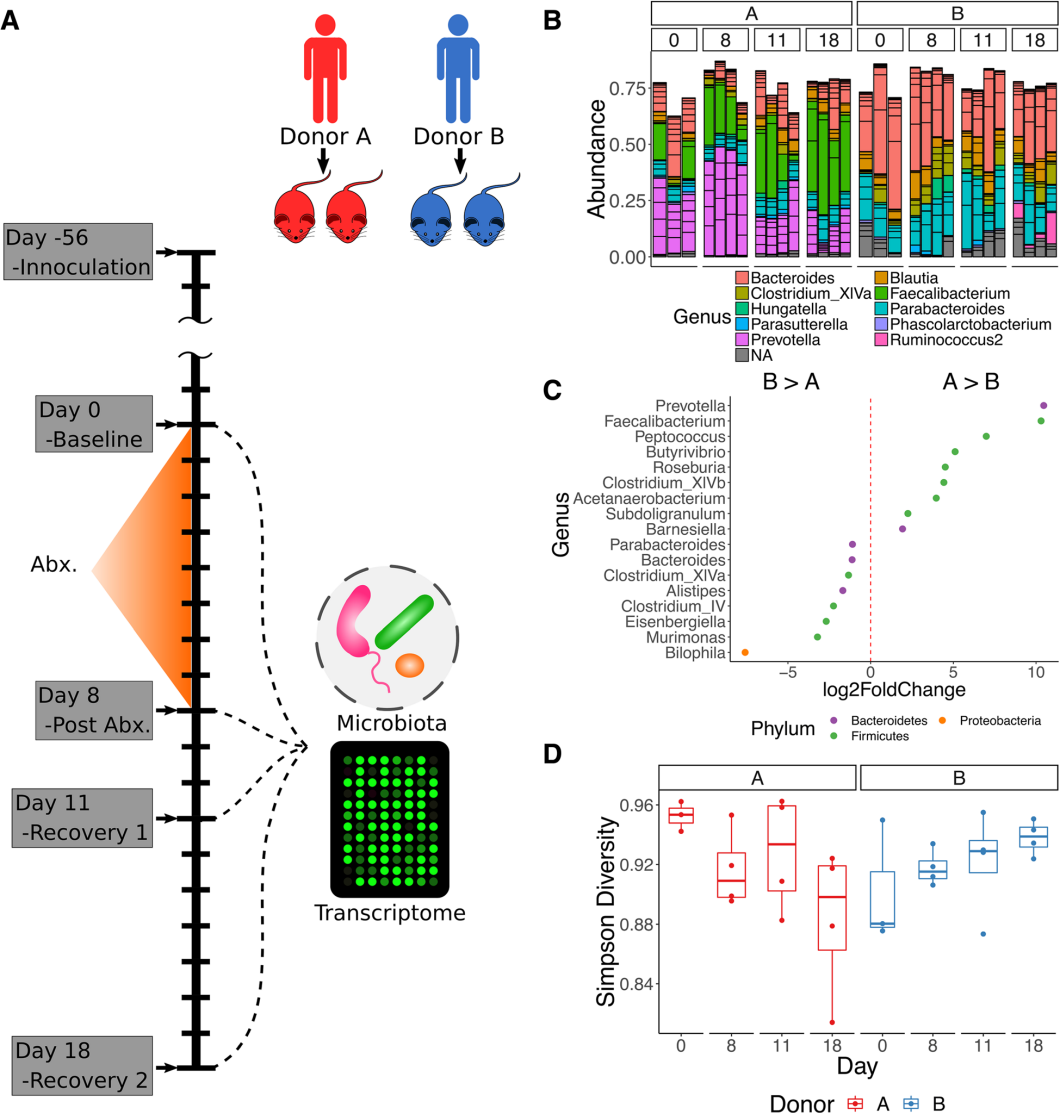
**a** Schematic of study design, including donor groups, antibiotic treatment, and recovery period as well as sampling points. **b** Barplots of relative genus-level abundance of the 50 most abundant OTUs in each sample. **c** Differentially abundant genera by DESeq2 between donor groups, including all time points. **d** Simpson diversity stratified by donor and time point

Due to the importance of the microbiota for host homeostasis and the marked collateral effects of antibiotics on the microbiota, we posed the questions: (a) can antibiotic administration result in correlated fluctuations of the microbiota and host colonic transcriptome? and (b) if so, is there evidence of a personalized response? To answer these questions, we characterized the microbiota and the colonic transcriptome of germ-free mice (GFM) humanized with fecal samples from two unrelated healthy human donors, before and after treatment with the broad-spectrum antibiotic amoxicillin-clavulanate (i.e., co-amoxiclav), commonly used to treat various infections in clinical practice (Fig. 1a).

## Results

In total, samples from 30 mice passed quality controls, 15 in each group. One animal from the donor B group at day 0 (D0) was excluded due to low microarray signal intensity values. Four hundred fifty-four OTUs (operational taxonomic units—see the “Methods” section for the definition as we used the *dada2* pipeline [22]), were identified following filtering and were assigned to 51 genera (mean sequences per sample following filtering 6089, range 4219–8835, rarefaction curves presented in Additional file 1: Figure S1A).

### Baseline microbiome composition modulates susceptibility and recovery following antibiotics exposure

Donor A recipient mice (donor A mice) had a markedly different community compared to mice from donor B. Donor A mice had a microbiota composition dominated by *Prevotella* (mean 26.6% (SD 13.6%)) and *Faecalibacterium* (mean 24.9% (SD 12.5%)), with *Bacteroides* (mean 17.3% (SD 7.8%)) being the third most dominant genus (Additional file 2). In donor B mice, the dominant genera were *Bacteroides* (mean 37.9% (SD 12.2%)) and *Parabacteroides* (mean 19.6% (SD 7.6%)), with less than 1% mean abundance of both *Faecalibacterium* and *Prevotella* (Fig. 1b, c). Samples for each donor were aggregated at each time point and submitted to enterotype clustering with the MetaHIT data set [23, 24] (following manual filtering to assure all genera matched) (Additional file 1: Figure S1B). This confirmed clustering of donor A mice with the *Prevotella* enterotype and donor B mice with the *Bacteroides* enterotype. Online reference-based enterotyping [25] similarly classified these samples to the *Prevotella* and *Bacteroides* enterotypes.

**Fig. 2:**
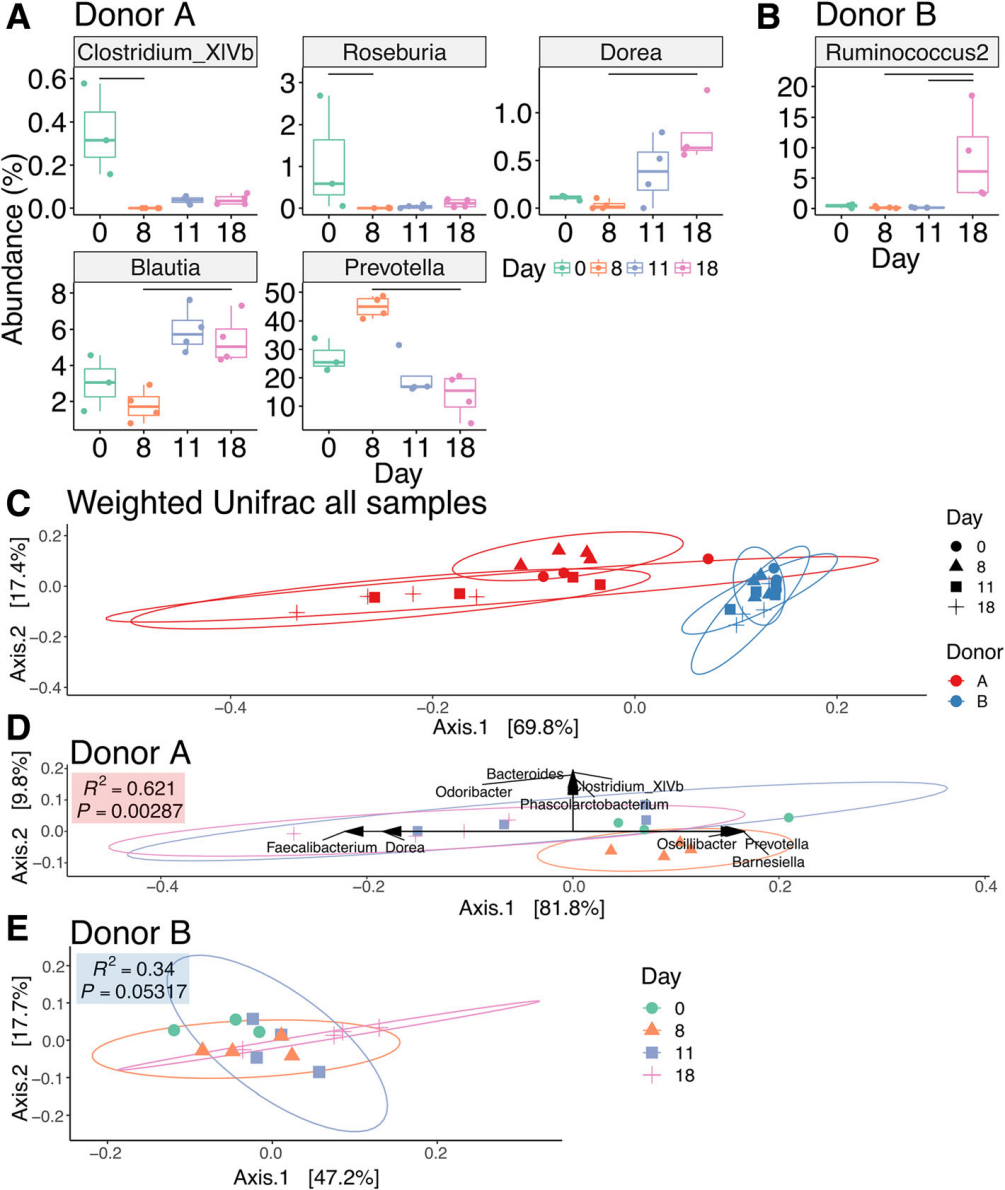
**a** Genera differentially abundant (adjusted *P* value < 0.01) in donor A mice by DESeq2. **b** Similar to **a** but for donor B mice. **c** Weighted unifrac distance with per group. Results of the corresponding two-way PERMANOVA are presented in Table 1. **d** PCoA of weighted unifrac distances for donor A alone, with genera that correlate significantly with PCoA axes (Spearman’s correlation, *P* value after correction 0.1) and PERMANOVA *R*^2^ and *P* value for univariate comparison. **e** PCoA for donor B as in **d** with weighted unifrac distances. No significant correlations were present. PERMANOVA *R*^2^ and *P* value for univariate comparison

The response to antibiotic administration was markedly different between the two donor groups. Immediately following co-amoxiclav treatment (D8—day 8), donor A mice demonstrated an increase in *Prevotella* sequences from baseline (D0) (from mean 27.4% (SD 5.9%) to mean 44.9% (SD 3.7%)) with a significant reduction in this genus by late recovery (mean 13.9% (SD 7.7%)) and a corresponding increase in *Faecalibacterium* OTUs at these time points (D0—mean 13.1% (SD 10.5%), D8—mean 21.3% (SD 5%), D11 (day 11)—mean 23.6% (SD 13%), D18 (day 18)—mean 38.5% (SD 7.4%); Fig. 1b, Additional file 2). There was a trend of decreasing proportional abundance in *Bacteroides* throughout the study in the donor B group, although this did not reach significance at any time point (D0—49.3% (SD 17.5%), D8—40.9% (SD 9.7%), D11—31.9% (SD 12%), D18—32.2% (SD 4%)), while *Parabacteroides* remained largely stable throughout (Additional file 2). Diversity was not significantly altered by antibiotic treatment (Fig. 1d, Additional file 3). Comparing different time points using DESeq2 for donor A mice, there was a significant reduction in *Clostridium* XIVb at D8 from baseline (D0), a significant reduction in *Roseburia* at D8 from D0, a significant reduction in *Prevotella* between D8 and D18, a significant increase in *Blautia* at D18 compared to D8, and a significant increase in *Dorea* at D18 compared to D8 (Fig. 2a).

In contrast to donor A mice, only one genus, *Ruminococcus*, increased significantly in donor B mice at D18 (Fig. 2b). Overall, there was a much more marked disruption of the community structure in mice from donor A mice when compared to donor B mice, evident in PCoA plots of weighted unifrac distance (Fig. 2c). To determine if there was a significant difference in terms of the effect of donor and day post antibiotics, we performed a two-way PERMANOVA, which indicated a significant interaction between donor and day (Table 1). A post hoc analysis demonstrated differences only for donor A, at pairwise contrasts between D0 vs D18, D8 vs D11, and D8 vs D18 (false discovery rate (FDR) *P* values < 0.05). Correlation of the PCoA scores with genera demonstrated a correlation between a number of key taxa, including *Prevotella* and *Faecalibacterium*, associated with the changes in donor A mice (Fig. 2d). No correlations were evident for donor B mice (Fig. 2e). While we did not have a no-antibiotic control group, we designed the study such that antibiotic-induced changes to the microbiota must occur independently in two cages. To ensure this was the case, we used a distance-based method to determine if within-cage differences over time (Additional file 4: Figure S2A) were significantly different from time point-specific distances, including between-cage effects at D8 (post antibiotics) (Additional file 4: Figure S2B and S2C). We observed a significant shrinking in the distance post antibiotics in donor A mice but did not observe a similar reduction in donor B mice, suggesting that the effects of antibiotics in this group were not above the baseline within-cage variability over the course of the study (Additional file 4: Figure S2C).

**Table 1:** Results of two-way PERMANOVA of weighted unifrac distances (random seed for rooting tree, 1234). *P* values (***< 0.001, *< 0.05)

	df	MeanSqs	*F* model	Post hoc test (FDR adjustment)
Main effects				
Donor	1	0.492	55.787***	
Day	3	0.0502	5.692***	
Two-way interaction				
Donor : Day	3	0.0236	2.674*	
				Contrasts Donor A: D0 vs D18* Donor A: D8 vs D11* Donor A: D8 vs D18*

Taken together, these results demonstrated a marked effect of co-amoxiclav administration on the microbiota of donor A mice, with notable shifts in the dominant genus *Prevotella* and a number of less abundant genera. In contrast, the microbiota profile of donor B mice remained relatively unaffected.

### Antibiotics induce dramatic changes on host transcriptome, which overlap independently of baseline microbiota composition

**Fig. 3:**
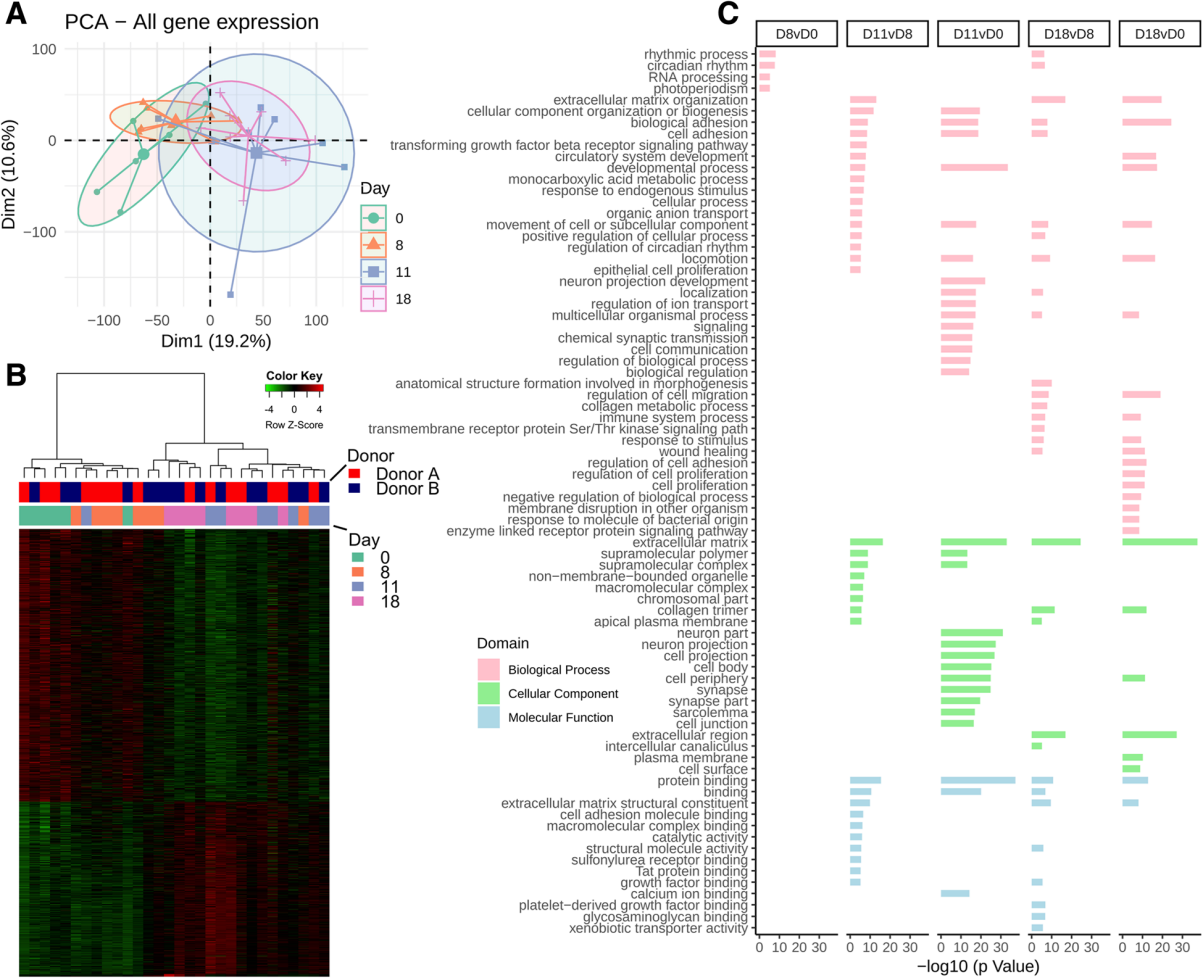
**a** PCA plots of gene expression data following antibiotic treatments for all mice combined, clustered by day with respect to antibiotic treatment. **b** Heatmap of differentially expressed genes at the D18 vs D0 contrast. **c** GO enrichment analysis, followed by submission to REVIGO, stratified by contrast

Gene expression was assessed initially with PCA for all mice combined (Fig. 3a). This demonstrated a pattern associated with antibiotic treatment, with the main separation between early (D0 and D8) and recovery (D11 and D18) time points. Figure 3b represents differentially expressed genes between D18 and D0 (1845 in total), which again demonstrate clear clustering between early (D0 and D8) and recovery (D11 and D18) time points. Gene Ontology (GO) analysis with the *goana* function in *limma* identified a large number of significant GO terms between different contrasts, so the top 100 were submitted to REVIGO and of the resulting output, those with a − log10(*P* value) > 5 were plotted. This analysis demonstrated significant changes in many terms, prominently biological processes concerned with extracellular matrix organization, cellular adhesion, signaling, cell migration, developmental processes, and circadian rhythm (Fig. 3c).

A combined analysis of both groups in our study also demonstrated significant differential gene expression alterations at D8 relating to genes associated with circadian rhythm, consistent with previously reported findings following antibiotic treatment, including altered expression of *Per* genes, *Cipc*, *Prkaa2*, *NR1D2*/*Rev-ErbA*, *Ciart*, *NPAS2*/*CLOCK*, *NFIL3*/*E4BP4*, and *ARNTL*/*BMAL1* [26] (Additional file 5: Figure S3). Recent work has precisely explored the relationship between circadian rhythm and the microbiome and identified diurnal biogeographic changes in microbial proximity to the epithelium which orchestrate epithelial and host circadian processes [27–29].

**Fig. 4:**
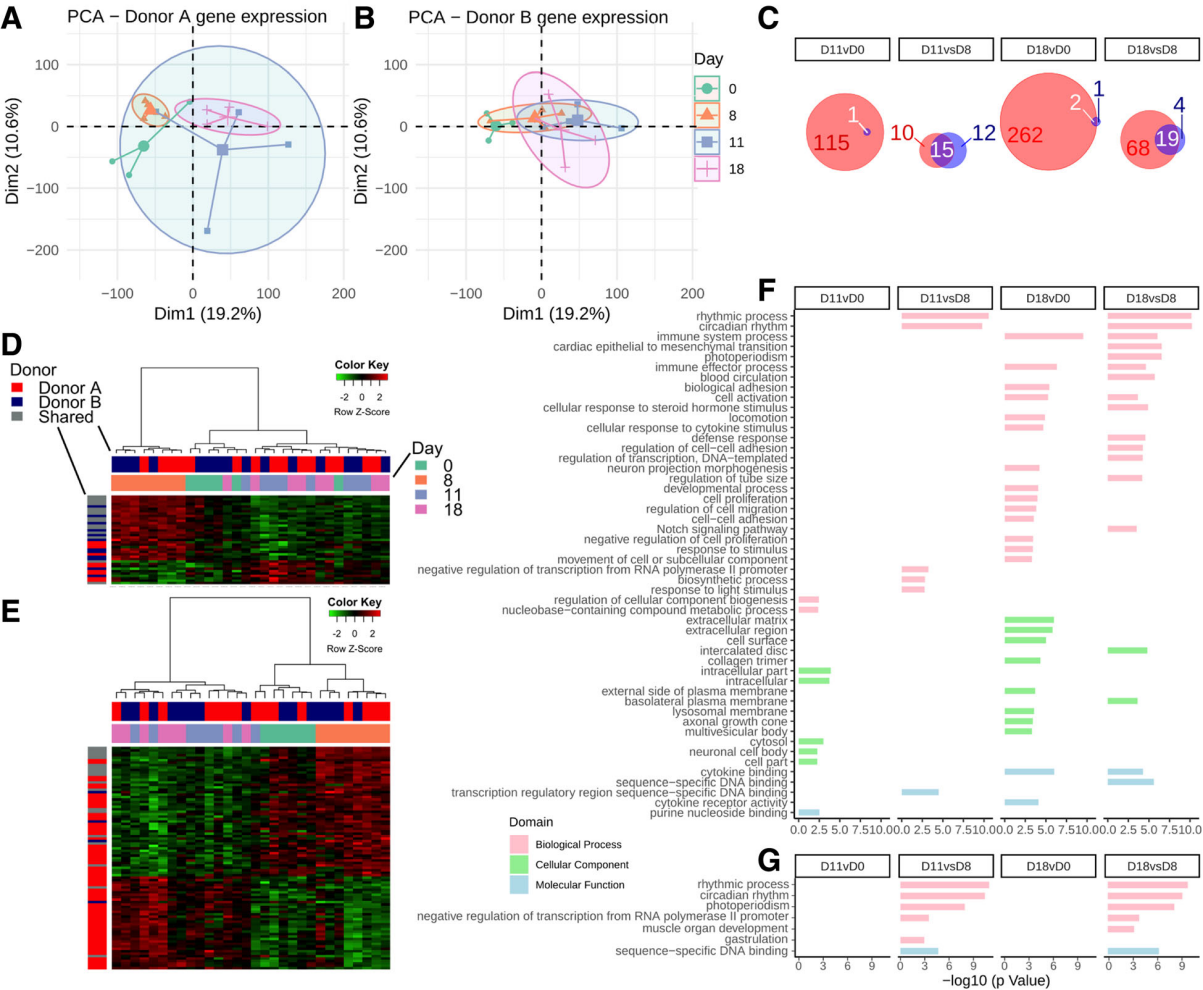
Gene expression analysis for both donor groups individually. **a** PCA as in Fig. 3a, isolating donor A gene expression. **b** PCA for donor B expression alone. **c** Contrasts with differential genes and the relative contribution from donor A and donor B. Red represents donor A, blue donor B, and white overlap. **d** D11 vs D8 heatmap of differential gene expression. **e** D18 vs D8 heatmap of differential gene expression. **f** GO enrichment analysis following submission to REVIGO, stratified by contrast for donor A. **g** As in **f** but for donor B

When the same analysis was repeated for each donor group individually, more significantly differentially expressed genes were detected in the donor A than donor B group (Fig. 4a–c). While only a limited number of differentially expressed genes were evident for donor B mice, there was notable overlap in those genes that were with donor A mice (Fig. 4c). Additionally, heatmaps of contrasts D11 vs D8 (Fig. 4d) and D18 vs D8 (Fig. 4e) including these genes, while dominated by donor A, still demonstrated clear clustering based on early (D0, D8) and late (D11, D18) time points, again suggesting that even though these genes did not meet the threshold for differential expression for donor B, there was a common pattern in response to co-amoxiclav. GO terms with at least four genes were submitted to REVIGO (donor A, Fig. 4f; donor B, Fig. 4g) demonstrated common activation of circadian rhythm terms at D8 compared to recovery time points (D11 and D18), while terms relating to immune system processes, cell adhesion and response to stimulus were evident in donor A mice at D18 when compared to the early time points (D0 and/or D8).

Taken together, these results demonstrate a marked effect of co-amoxiclav administration on the colonic transcriptome of humanized mice. Gene expression analysis identified changes in genes involved in extracellular matrix organization, cellular adhesion and motility, developmental processes, circadian rhythm, and immune system processes. While the pattern of gene expression was similar regardless of the baseline (D0) microbiota, the degree of change appeared to be more marked in mice humanized by donor A than by donor B. As these were the mice that had a more marked alteration in the microbiota composition in response to co-amoxiclav, we next investigated whether correlation between the transcriptome and microbiota was evident.

### Host transcriptome-microbiota correlation reveals marked covariance between the microbiota and GO pathways in donor A mice but not in donor B mice

**Fig. 5:**
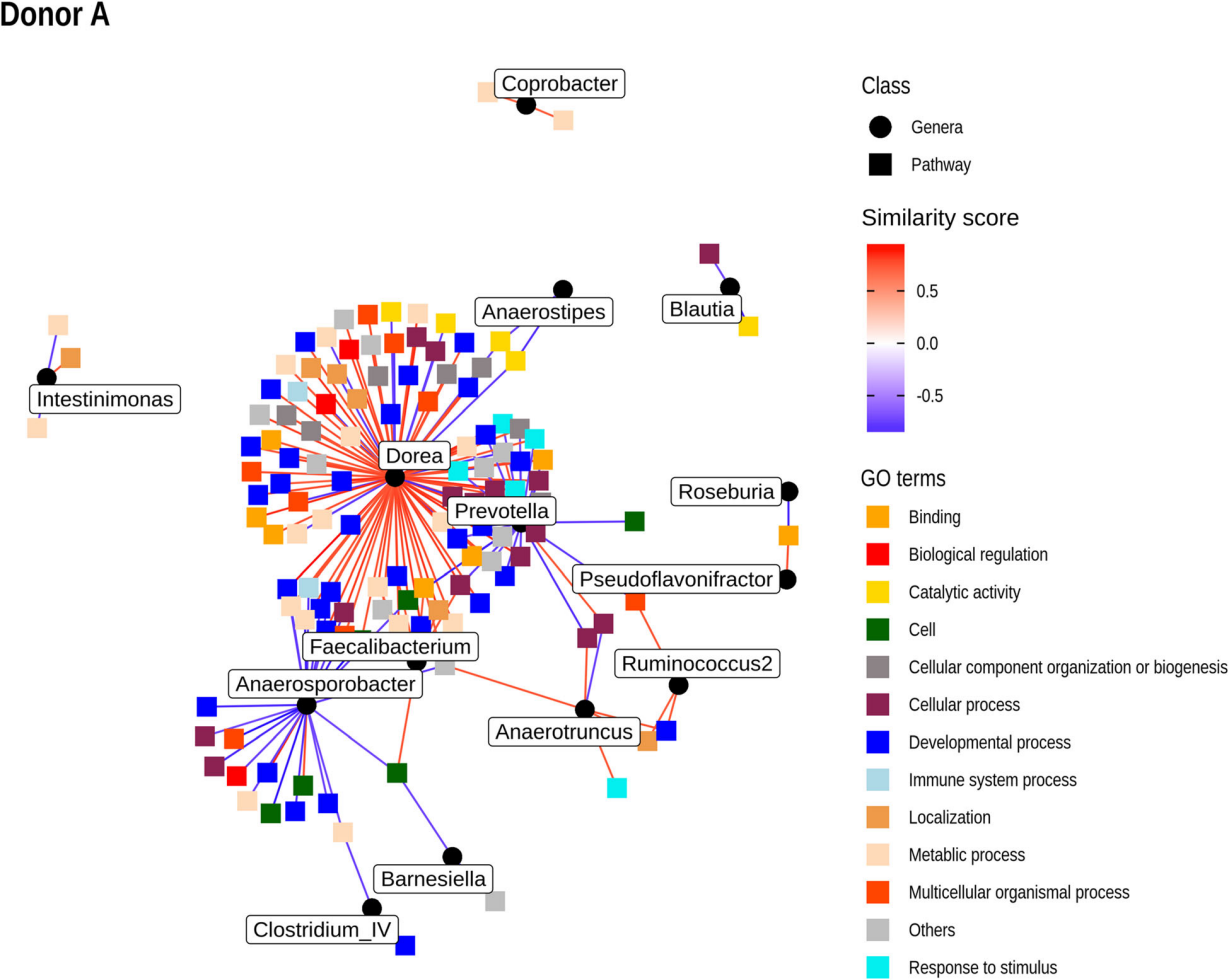
Network plot of significant correlations between GO pathways drawn from GSVA and genera abundance for donor A mice determined by the HAllA method. Corresponding GO pathways are provided in Additional file 6

Due to the large number of differentially expressed genes across the different contrasts, we used Gene Set Variation Analysis (GSVA) to identify gene pathways associated with antibiotic treatment. To perform correlation with microbial taxa, we used Hierarchical All-against-All significance testing [30], a recently developed technique for determining relationships between multi-omics datasets (Fig. 5, Additional files 6 and 7). This method identifies significant correlations between individual features of both datasets, as well as identifying correlations between clusters of features. Performed using Spearman’s correlation values on the genus-level abundance from each donor and the corresponding pathway abundance from GSVA, a large number of correlations were evident for donor A mice following multiple-hypothesis correction, including a number of dominant and/or differentially abundant bacterial taxa from that donor (*Prevotella*, *Dorea*, *Faecalibacterium*, *Blautia*, and *Roseburia*). Figure 5 represents a network plot of significant correlations between individual pathways and bacterial genera for donor A mice. The pathways are colored by high-level GO assignments. Furthermore, pathway-bacteria correlations, including additional clusters determined by HAllA, are represented in Additional file 6 (data) and Additional file 7: Figure S4. In contrast, no significant correlations between pathway expression and the microbiota were evident for donor B mice.

Taken together, these results demonstrate correlation between host pathways and microbiome composition was unique to donor A mice. However, as the antibiotic-induced changes in donor B mice were considerably weaker, such correlations are harder to detect and higher numbers of mice would probably be required in this case.

## Discussion

We report that GFM humanized with different human donor microbiota maintain a unique community profile and experience individualized responses to oral antibiotic administration. Mice humanized with donor A microbiota were dominated by *Prevotella* and *Faecalibacterium*, with a lesser contribution from *Bacteroidetes* and underwent marked fluctuations in terms of their dominant genera, most prominently *Prevotella* and *Faecalibacterium*, as well as in other lower abundance genera within the community. It is notable that by D18, the mean abundance of *Prevotella* was approximately half that of baseline (D0), while the mean proportional abundance of *Faecalibacterium* was almost three times the baseline (D0) (Fig. 1b and Additional file 2), suggesting that the donor A group had a lower resilience, without return to baseline by the end of the study.

In contrast to donor A, donor B mice were much more resilient to antibiotic administration, with the notable exception of a late bloom in *Ruminococcus* sequences at D18. *Bacteroides* was the dominant genus in this group with *Parabacteroides*, the second most dominant genus. As mentioned, there was a trend of decreasing proportional abundance in *Bacteroides* throughout the study in the donor B group, although this did not reach significance. Notably, most dramatic changes in microbiota composition occurred in the recovery period.

We did not observe a significant change in *α*-diversity over the duration of the study. We note that there were only three baseline (D0) mice in each group and this may have accounted for our inability to detect a significant change. Furthermore, as we did not detect changes in general in the donor B group, it is consistent that we did not detect a change in *α*-diversity either. In terms of donor A mice, we did note a drop-off in the Simpson diversity at D18 (Fig. 1d) but changes in this donor group appeared to be dominated by compositional changes rather than a reduction in richness (unique OTUs).

Marked effects of antibiotics on the colonic transcriptome were also demonstrated, again most notably in the recovery period. In a pooled analysis, there was a significant increase in many processes related to biological adhesion and sensing (Fig. 3), perhaps due to stimulation from a rebound recovery in bacteria following the withdrawal of antibiotics. When analyzed individually, the gene expression changes were more marked for donor A mice, suggesting that the more dramatic perturbations in composition associated with this donor group were reflected in the transcriptome. Stronger evidence for this conclusion can be drawn from the multiple correlations detected between enriched GO pathways and the microbiota composition of donor A mice (Fig. 5), in contrast to those of donor B. While these correlations only represent an association, it must also be noted that the total bacterial load was not quantified in this study and as such, the changes in composition may reflect significant changes in mucosal-associated bacteria, which were perhaps more dramatically altered in donor A mice.

Recently, a study has elegantly elucidated the effects of an ablative cocktail of antibiotics on the colonic transcriptome, identifying that depletion of the microbiota, direct antibiotic effects on the host, and the effects of antibiotic-resistant microbiota account for the alterations [31]. This study also demonstrated downregulation of immunity with antibiotic treatment, while identifying significant direct toxicity of antibiotics on mitochondrial function. Interestingly, a recent study in healthy adults using a cocktail of antibiotics demonstrated dramatic, rapid shifts in microbiome composition, followed by near-complete recovery within 6 months [32]. In contrast, our study utilized a more gentle perturbation with a single antibiotic combination commonly used in human medicine, in order to determine correlations between disturbances in the microbiota and the transcriptome, in addition to their relationships to baseline microbiota composition.

Our data suggest that while the response to antibiotics is very different between groups of GFM humanized by different donors, and therefore enterotypes, gene expression patterns in response to antibiotics are similar. However, the magnitude of the change detected was more evident in the donor A group, suggesting that the magnitude of microbiota perturbation influences the subsequent changes in gene expression. Such a conclusion fits with numerous observations in murine models highlighting the importance of the baseline microbiota, its impact in a range of settings, and the requirement for methods to quantify and standardize microbiome effects in animal models [33]. These data also suggest that in multi-omics studies incorporating the microbiome, temporal analysis within individuals may yield more insights than aggregating data between individuals with very different baseline microbiota compositions. This was clearly evident in our results, where aggregation of data led to a complete loss of the covariance structure due to the different levels and responses of shared bacterial genera between donor groups. These findings would also fit more broadly with emerging data indicating that our microbiome represents an important element of our personalized response to environmental factors [34, 35].

A number of limitations should be noted in our study. Only two donors groups were available for analysis and therefore findings relating to the behavior of individual genera, both in their response to antibiotics and their association with host transcriptome response, cannot necessarily be extrapolated to include a range of donor microbiome compositions. Furthermore, varying doses of antibiotics, administration protocols, and antibiotic cocktails have been used in previous studies, limiting direct comparisons. It is important to note that we did not sequence the original donor communities and therefore we cannot characterize precisely the true extent of engraftment of strains or the resulting compositional similarity to the original fecal sample. We have based our conclusions on this model protocol as it is established in our facility in multiple studies that replicate phenotypic traits of the donor [36–39], as well as from other groups, where seminal findings have established that up to 85% of human taxa can be established following oral gavage [40]. However this study and other similar studies have used higher initial concentrations, and other studies have used higher quantities again [41], as well as repeated dosing schedules [42]. We do however note that the microbiota communities in our study clustered closely with human donors from the MetaHIT consortia, suggesting we had recapitulated an adequate proportion of the donor microbiota in each group. In addition to these specific points in relation to our study, humanized GFM also suffer from a number of limitations, including the effects of a non-native microbiota and impaired immune development in the humanized mouse intestine and these must also be acknowledged [43].

In addition, we did not use a no-antibiotic control group but instead housed mice in one of two separate cages for each donor group. This design implicitly required that changes be consistent across cages in each donor group. We acknowledge however that this represents a limitation in our study. We have used the cage effects in the study to demonstrate significant convergence of microbiota communities in donor A post antibiotics (D8) and not in donor B. However, conclusions from both the microbiota and host transcriptome must take into account the lack of a no-antibiotic control group.

Finally, recent work has demonstrated the importance of bacterial load in characterizing the microbiome, and changes in total bacterial counts in both donor groups may have contributed to some of the effects seen [44].

## Conclusions

In summary, we have demonstrated that a commonly prescribed antibiotic can exert significant effects on the gut microbiota, in concert with colonic gene expression. Notably, while the host response appeared to be conserved, the degree of activation was related to the degree of microbiota perturbation, resulting in correlations between host transcriptome and the microbiota unique to one donor community. These findings offer a window into the role that our microbiome plays in mediating our personalized response to medications such as antibiotics.

## Materials and methods

### Study design

All procedures in relation to the care and use of laboratory animals were carried out in accordance with European guidelines and the local ethics committee approved this study. Germ-free male 6-week-old mice (*n* = 31) were transferred into two sterile separate isolators (Table 2; ANAXEM, INRA, Jouy-en-Josas, France) for the 10-week duration of the study and had ad libitum access to irradiated feeds and sterile water. Mice were observed once a day to ensure their welfare. For colonization, GFM were inoculated orally with 250 μl of a 10^−2^ dilution of whole fecal homogenate from two healthy adults (termed donors A and B) with no history of intestinal problems or intake of antibiotics 3 months prior to the beginning of the study, resulting in a single donor fecal microbiota transplantation per isolator (donor A (*n* = 15) and donor B (*n* = 16)). Mice in each isolator were housed in two separate cages (Table 2), with the aim to have four mice per time point (two per cage) following antibiotics in each group. The human donors who provided a fecal sample gave their verbal consent to do so. Fifty-six days after microbiota inoculation, samples were acquired at baseline (day 0 (D0); donor A (*n* = 3), donor B (*n* = 4)), followed by the administration of the penicillin antibiotic amoxicillin in combination with the *β*-lactamase inhibitor clavulanic acid once a day (80 mg and 10 mg/kg) to all remaining mice. We chose this antibiotic as it is a commonly prescribed, broad-spectrum human antibiotic, and treatment was continued for a total of 7 days, similar to the duration of a typical treatment for a community infection [45], followed by sampling at days 8 (D8), 11 (D11), and 18 (D18) (Fig. 1a). We choose this dose as it is intermediate between doses used in previous rodent studies [46, 47] and is the current recommended dosing for children under 40 kg body weight in France [48].

**Table 2:** Number of samples according to time points, donors, and cages

Donor	Cage	D0	D8	D11	D18
A	1	2	2	2	2
A	2	1	2	2	2
B	1	2	2	2	2
B	2	2	2	2	2

### Sampling

At each time-point, a subset of animals was sacrificed (see Table 1), the large intestine (colon) was removed and luminal contents acquired and processed for the extraction of community microbial DNA. RNA extraction proceeded directly from the large intestine. We did not have prior data to estimate the duration of the recovery period to monitor and so early recovery (D11, 3 days post-cessation of antibiotics) and late recovery (D18, 10 days post-cessation of antibiotics) time points were chosen.

### Gene expression

Total RNA was isolated from the large intestine with the RNeasy Mini Kit (Qiagen, Courtaboeuf, France) according to the manufacturer’s instructions. RNA 6000 Nano chips on a Bioanalyzer 2100 (Agilent Technologies, Les Ulis, France) were used to assess RNA integrity. Gene expression profiling was performed with the SurePrint G3 Mouse GE 8x60 K Microarray (Design ID: 028005, Agilent Technologies). Further details are provided in supplementary methods in Additional file 8.

### DNA extraction and sequencing

Fecal samples were weighed and resuspended in 250 μl of 4 M guanidine thiocyanate in 0.1 M Tris at pH 7.5 (Sigma) and 40 μl of 10% *N*-lauroyl sarcosine (Sigma) for 10 min at room temperature. Five hundred microliters of 5% *N*-lauroyl sarcosine in 0.1 M phosphate buffer (pH 8.0) was then added and incubated at 70 °C for 1 h. A mixture of sterilized silica beads (0.1- and 0.6-mm diameter) was added and the tube shaken three times at 6.5 m/s for 30 s in a FastPrep apparatus (MP Biomedicals). Fifteen milligrams of polyvinylpolypyrrolidone was then added to the tube, and this was vortexed and centrifuged at 20,000×*g* for 5 min. Following the recovery of the supernatant, the pellets were washed with 500 μl of TENP (50 mM Tris (pH 8), 20 mM EDTA (pH 8), 100 mM NaCl, 1% polyvinylpolypyrrolidone) and centrifuged for 5 min at 20,000×*g* and the new supernatant was added to the first supernatant. The washing step was repeated twice. The pooled supernatant was centrifuged briefly to remove particles and then split into two 2-ml tubes. Precipitation of nucleic acids was performed by adding one volume of isopropanol for 10 min at room temperature with centrifugation at 20,000×*g* for 10 min. Pellets were then resuspended and pooled in 450 μl of 100 mM phosphate buffer at pH 8 and 50 ml of 5-M potassium acetate. The tube was placed on ice overnight and then centrifuged for 30 min at 20,000×*g*. The supernatant was subsequently transferred to a new tube containing 20 μl of RNase (1 mg/ml) and incubated at 37 °C for 30 min. Nucleic acid precipitation was performed by adding 50 μl of 3-M sodium acetate and 1 ml of absolute ethanol. The tube was incubated for 10 min at room temperature and the nucleic acids were recovered by centrifugation at 20,000×*g* for 15 min. The resulting DNA pellet was finally washed with 70% ethanol and then dried and resuspended in 100 μl of Tris–EDTA (TE) buffer.

Amplicon libraries for barcoded 454 pyrosequencing were generated by PCR of the V3–V4 hypervariable region of the 16S gene with the primers 343F 5′-TACGGGAGGCAGCAG-3′ and 806R 5′-GGACTACCAGGGTATCTAAT-3′. Barcodes are available in the supplementary methods (Additional file 8).

### Bioinformatic and statistical analysis

#### Transcriptome analysis

Following the pre-processing of raw transcriptome data (described in detail in Additional file 8), statistical analysis was performed in the R statistical environment (R version 3.6.0) [49].

#### Differential gene expression analysis

Principal component analysis was performed using the ade4 (v1.7-13) [50] and factoextra (v1.0.5) packages in R. Differential gene expression was performed using the *limma* package (v3.36.5) and the eBayes test [51]. Multiple testing corrections were made using the Benjamini-Hochberg method (adjusted *P* values < 0.05). Differentially enriched pathways in the Gene Ontology (GO) databases were identified using the *goana* function in the *limma* package. To remove redundancy, GO terms that were significantly enriched (adjusted *P* value < 0.01) were submitted to REVIGO [52] and the output presented by way of – log10 (*P* value).

#### Microbiome analysis

Demultiplexing of pyrosequencing fasta and quality files was performed with QIIME (v1.9.1) [53] and FASTQ files were created which were loaded into R and analyzed using the DADA2 package (v1.10.0), optimized for pyrosequencing output [54] (see Additional file 8) to give a table of amplicon sequence variants (referred to as operational taxonomic units (OTUs) throughout). Taxonomic assignments were performed using the RDP v16 (Ribosomal Database Project) training set specially formatted for the DADA2 package [55]. Phylogenetic tree construction utilized the DECIPHER [56] and Phangorn [57] packages. All subsequent microbiome analyses were performed through the phyloseq (v1.24.2) and vegan (v2.5-5) packages in R [22, 58, 59].

Alpha diversity was presented using the Simpson diversity index. Differential abundance between donor groups and between different time points within donor groups was assessed using *DESeq2* (v1.24.0) (FDR *P* values < 0.01) and significance was confirmed by Mann-Whitney testing [60]. Beta diversity was assessed with principle coordinate analysis (PCoA) of weighted unifrac distances [61] on proportional abundance data (proportion normalized). A two-way PERMANOVA was performed, using donor and day as main effects and the interaction between donor and day as the interaction term. Post hoc pairwise testing was performed using the “pairwise.perm.manova” function from the *RVAideMemoire* package (v 0.9-73) [62]. Spearman’s correlation of genus-level microbial taxa was also performed against the first two principal coordinate axes for each donor group individually to illustrate bacterial genera associated with major variation. *P* values were adjusted with the Benjamini-Hochberg method and correlations with adjusted *P* values < 0.1 were retained. Graphs were made using *ggplot2* (v3.1.1) [63] (Additional file 9).

#### Correlation of microbiota and transcriptome

To determine if there was evidence of the correlation between antibiotic-induced changes in the microbiota and the transcriptome, we firstly used Gene Set Variation Analysis (GSVA) to identify enriched GO pathways across the entire transcriptome data set, using the R package GSVA (v1.32.0) [64]. GO pathways were derived from a murine version of the Molecular Signatures Database (MSigDB) [65]. GSVA output was then submitted to *limma* to determine differentially expressed pathways. This allowed for dimensionality reduction, reducing the total number of transcripts (18,217) and differentially expressed genes (3630) to 182 differentially expressed pathways across all contrasts. Due to the extreme differences in microbiota composition between the two donor groups, pathway scores were correlated with genus-level bacterial abundance for each group individually. Correlation was performed using the Hierarchical All-against-All (HAllA) approach developed for multiomics data sets [30], having discarded pathways with log fold changes of < 0.5. Benjamini-Hochberg-adjusted *P* values of < 0.1 were retained from the results and were plotted using *ggraph* and *igraph* [66] (Additional file 10).

## Additional files

Additional file 1: **Figure S1.** A. Rarefaction curves for 16S sequences per sample. Observed species and Shannon diversity are presented. B. Data from MetaHIT and this study submitted to enterotyping with mean abundance at each time point for each donor group, demonstrating clustering of donor A with the *Prevotella* enterotype and donor B with the *Bacteroides* enterotype. (TIFF 1269 kb)

Additional file 2: Tables of proportional abundance of different genera in the different donor groups. (XLSX 18 kb)

Additional file 3: Results from statistical tests of significance for different diversity metrics. (XLSX 8 kb)

Additional file 4: **Figure S2.** Distance-based analysis examining the effects of antibiotics within and between cages. Schematic of the weighted unifrac distances between time points within individual cages for a donor group (S2A) and the distances at individual time points, including those between cages (S2B). In S2C, these distances are plotted for both donor groups, indicating a significant shrinking in distance post antibiotics (D8) in donor A mice, while there is no significant difference for donor B mice. (TIFF 1150 kb)

Additional file 5: **Figure S3.** Heatmap of differentially abundant genes relating to the circadian rhythm at the contrasts for both donor groups (all mice) combined. (TIFF 943 kb)

Additional file 6: Output from HAllA correlations of GSVA and genus abundance. (XLSX 43 kb)

Additional file 7: **Figure S4.** Heatmap output from Hierarchical All-against-All significance procedure for donor A. (PDF 138 kb)

Additional file 8: Supplementary methods and pyrosequencing barcodes. (DOCX 118 kb)

Additional file 9: Output describing the code used for microbiota analysis. (HTML 3490 kb)

Additional file 10: Output describing the code used for transcriptome analysis. (HTML 2420 kb)

## Data Availability

Microarray data is available from the Gene Expression Omnibus (GEO) with accession number GSE131785, while microbiota sequencing data is available from the Sequence Read Archive (SRA) with accession number PRJNA544904. Files detailing the code used for the analysis are available as R knitr.html files. For the microbiota analysis, this is in Additional file 9, and for the transcriptomic analysis, this is in Additional file 10.

## Abbreviations

CDI: *Clostridium difficile* infection
DNA: Deoxyribonucleic acid
EDTA: Ethylenediaminetetraacetic acid
FDR: False discovery rate
GFM: Germ-free mice
GO: Gene Ontology
GSVA: Gene Set Variation Analysis
HAllA: Hierarchical All-against-All significance testing
MsigDB: Molecular Signatures Database
OTU: Operational taxonomic units
PCA: Principal component analysis
PCoA: Principal coordinate analysis
PCR: Polymerase chain reaction
RNA: Ribonucleic acid
VRE: Vancomycin-resistant *Enterococcus*
