# Supplementary figures and images for: Baseline microbiota composition modulates antibiotic-mediated effects on the gut microbiota and host
Source: Microbiome. 2019 Aug 2;7:111. doi: 10.1186/s40168-019-0725-3 (PMC6676565; doi:10.1186/s40168-019-0725-3)

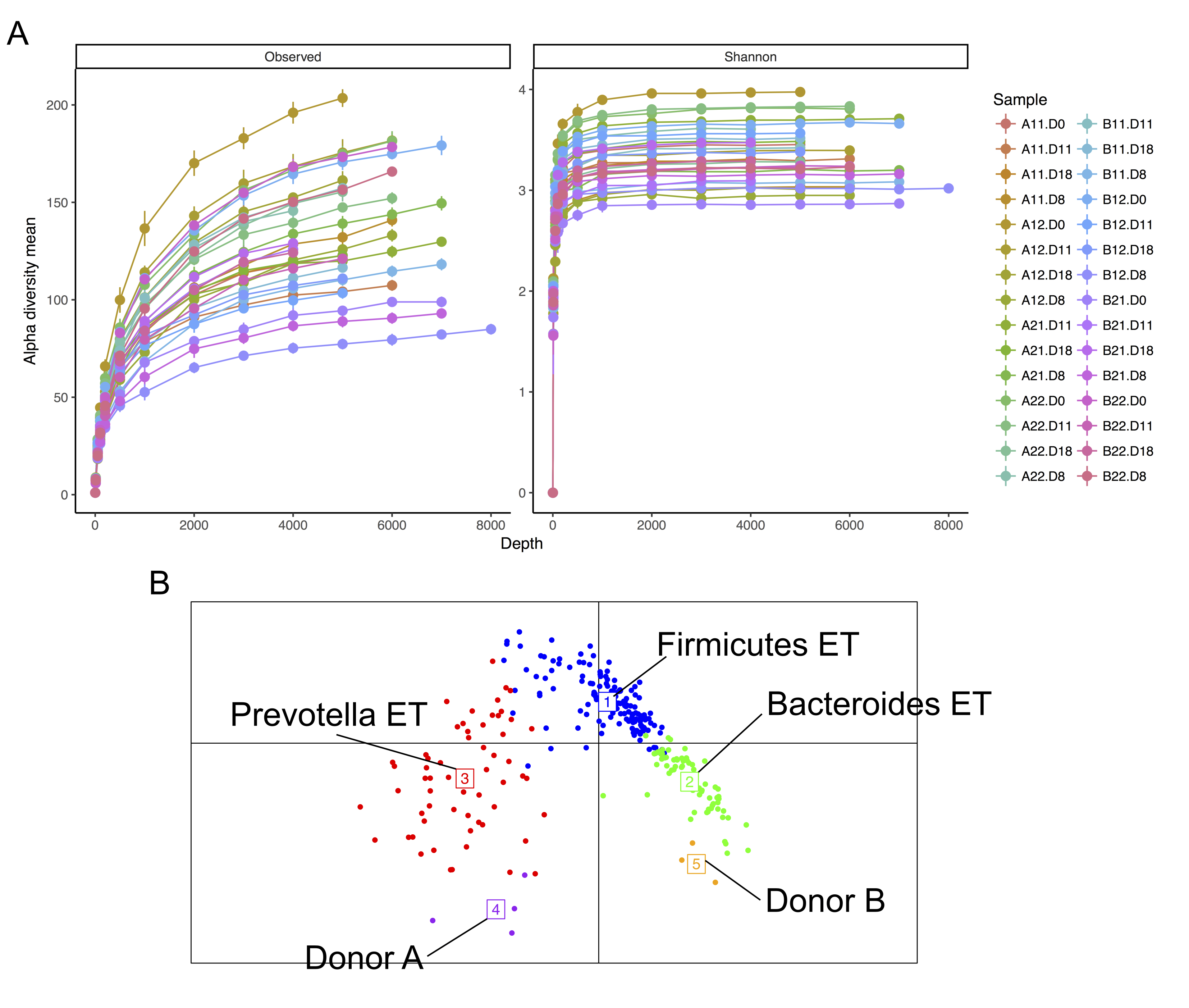

Supplement: Supplementary file 1 — Figure S1. A. Rarefaction curves for 16S sequences per sample. Observed species and Shannon diversity are presented. B. Data from MetaHIT and this study submitted to enterotyping with mean abundance at each time point for each donor group, demonstrating clustering of donor A with the Prevotella enterotype and donor B with the Bacteroides enterotype. (TIFF 1269 kb) [file 40168_2019_725_MOESM1_ESM.tiff]

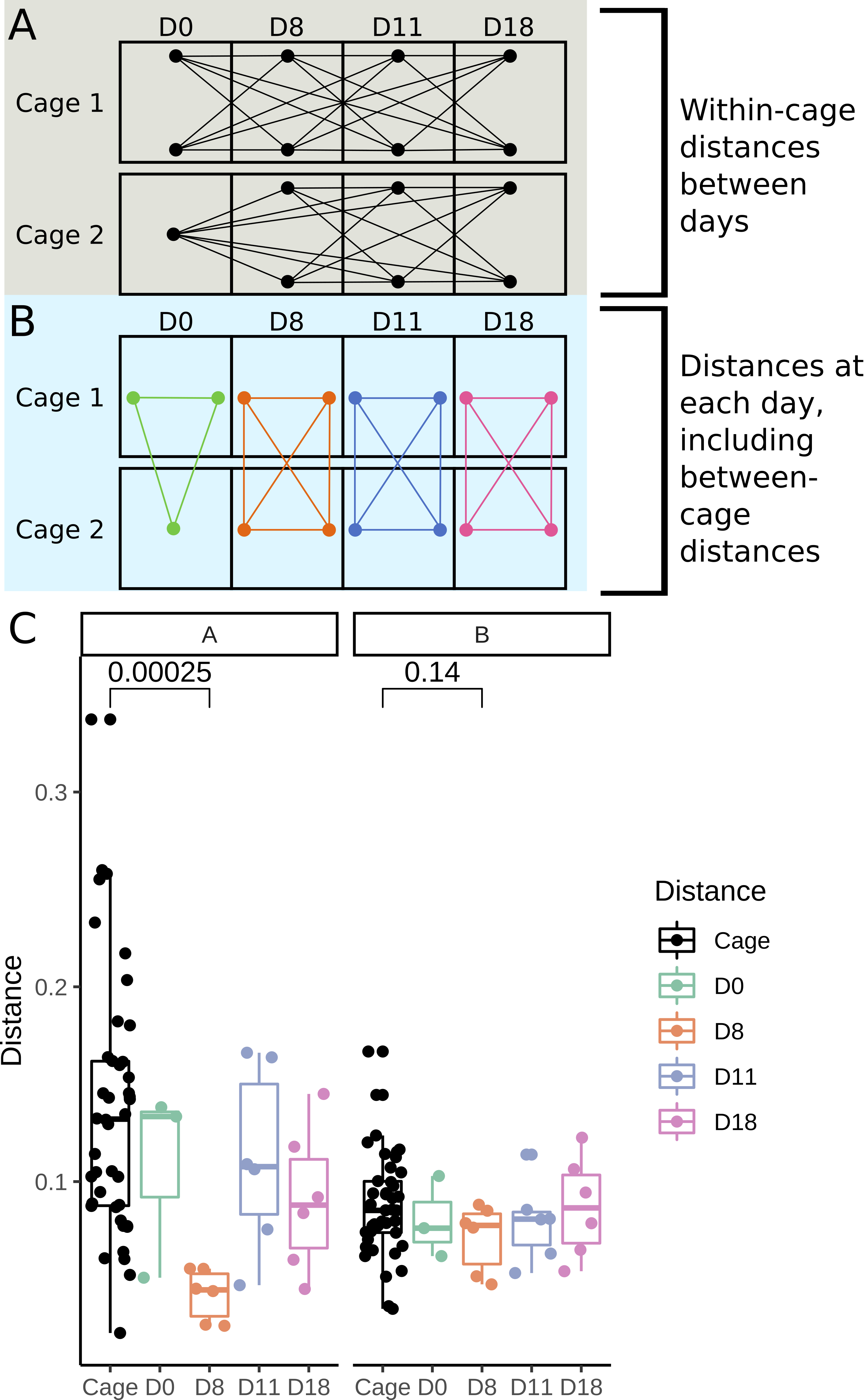

Supplement: Supplementary file 4 — Figure S2. Distance-based analysis examining the effects of antibiotics within and between cages. Schematic of the weighted unifrac distances between time points within individual cages for a donor group (S2A) and the distances at individual time points, including those between cages (S2B). In S2C, these distances are plotted for both donor groups, indicating a significant shrinking in distance post antibiotics (D8) in donor A mice, while there is no significant difference for donor B mice. (TIFF 1150 kb) [file 40168_2019_725_MOESM4_ESM.tiff]

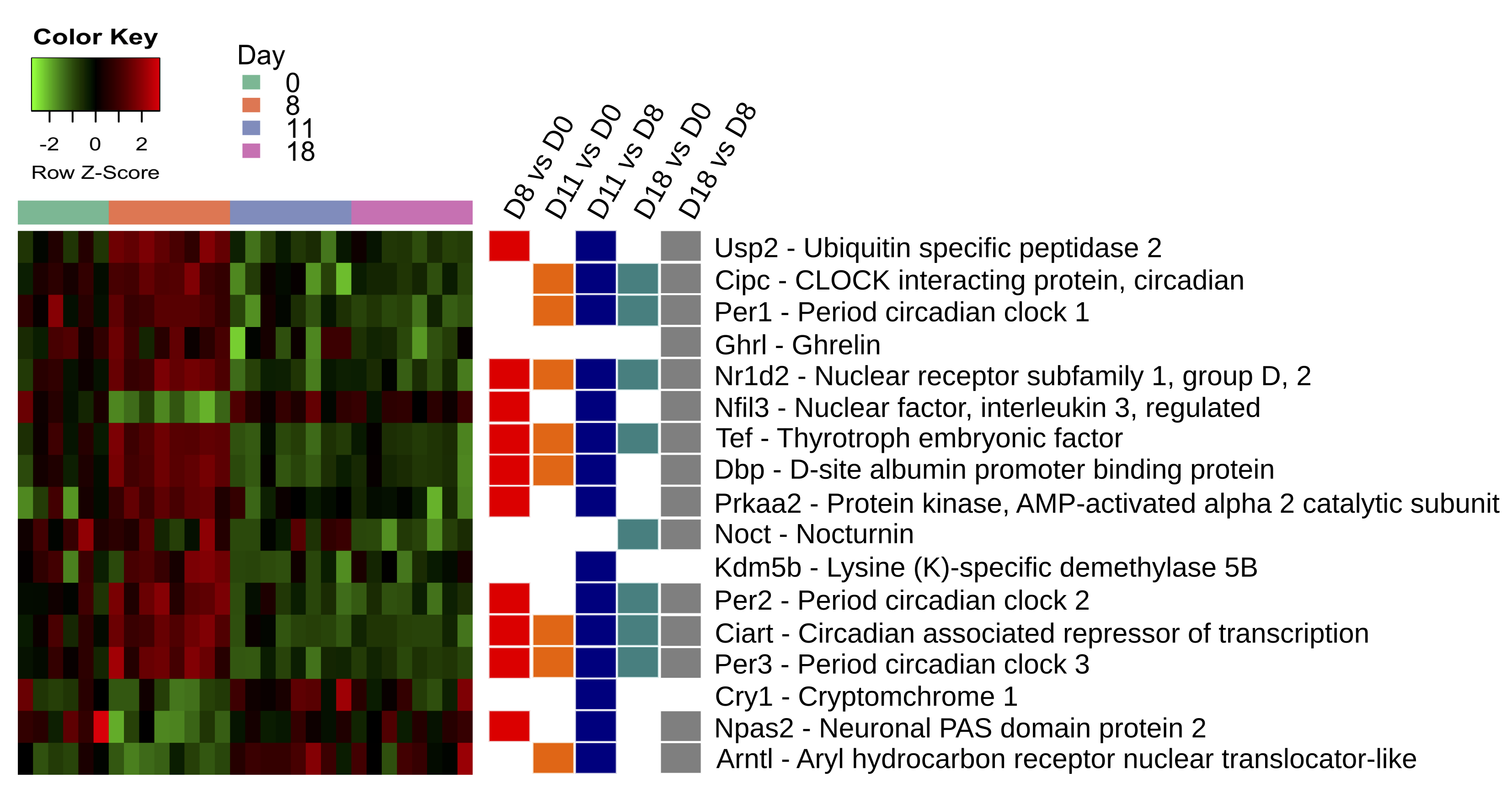

Supplement: Supplementary file 5 — Figure S3. Heatmap of differentially abundant genes relating to the circadian rhythm at the contrasts for both donor groups (all mice) combined. (TIFF 943 kb) [file 40168_2019_725_MOESM5_ESM.tiff]

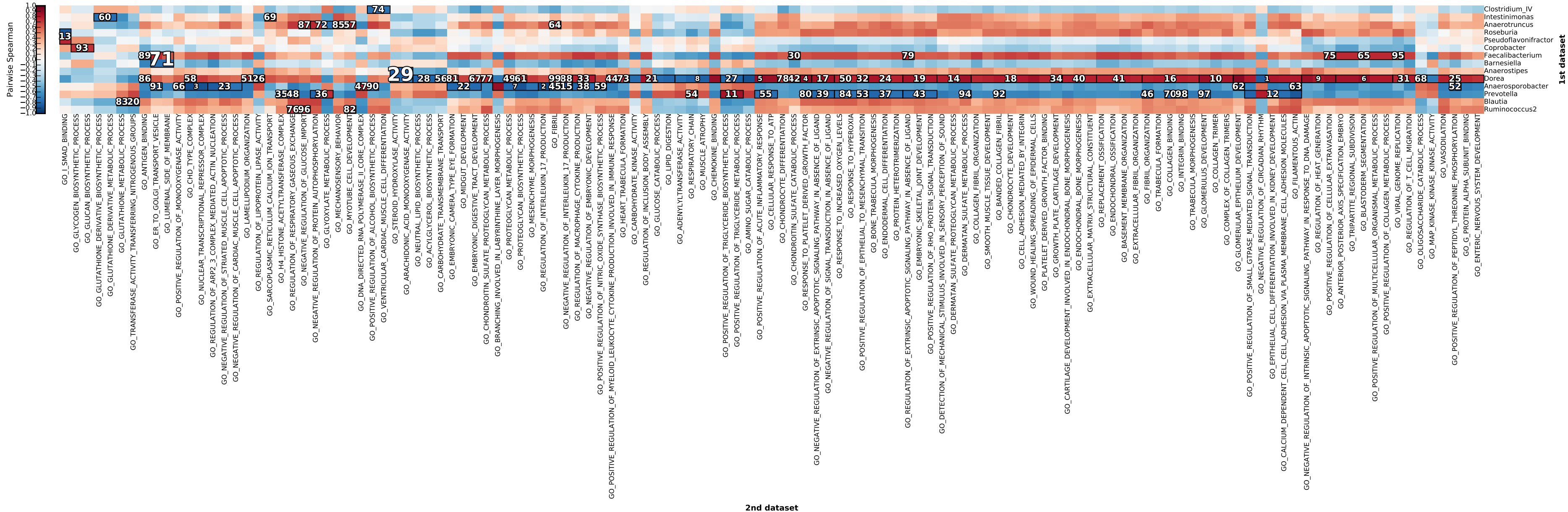

Supplement: Supplementary file 7 — Figure S4. Heatmap output from Hierarchical All-against-All significance procedure for donor A. (PDF 138 kb) [file 40168_2019_725_MOESM7_ESM.pdf]
