# Supplementary material for: Baseline microbiota composition modulates antibiotic-mediated effects on the gut microbiota and host
Source: Microbiome. 2019 Aug 2;7:111. doi: 10.1186/s40168-019-0725-3 (PMC6676565; doi:10.1186/s40168-019-0725-3)
Supplement: Supplementary file 8 — Supplementary methods and pyrosequencing barcodes. (DOCX 118 kb) [file 40168_2019_725_MOESM8_ESM.docx]

**Supplementary methods**

**Gene expression**

The SurePrint G3 Mouse GE 8x60K Microarray (Design ID: 028005, Agilent Technologies) was used for transcriptional analysis. Cyanine-3 (Cy3) labeled cRNAs were prepared with 100 ng of total RNA using the One-Color Low Input Quick Amp Labeling kit (Agilent Technologies) and following the recommended protocol. cRNA yields were determined using a NanoDrop ND-1000 (Thermo Fisher Scientific). 600 ng of Cy3-labeled cRNA per sample (specific activity > 11.0 pmol Cy3/µg of cRNA) was fragmented for 30 minutes at 60°C. This was followed by hybridization at 65°C to the microarray for 17 hours in a rotating hybridization oven (Agilent Technologies). Subsequently, microarrays were washed and dried. A G2565CA Scanner System (Agilent Technologies) was used to scan the slides. TIFF images were output and analyzed Feature Extraction Software v10.7.3.1 (Agilent Technologies) was used to analyse them, using the GE1_107_Sep09 protocol.

**Microarray analysis**

***Pre-processing data***

The Agilent Feature Extraction Software v10.7.3.1 was used to convert scanned signal into tab-delimited text files, which could be analyzed by third-part software. Raw data was imported into the R statistical environment [1]. Boxplot and principal component analysis (PCA) of raw data and transformed data were used to get an overview of the data using the ade4 package [2]. At this stage, one sample from the donor B mice group (Day 0), was excluded due to low signal intensity.

P-values output by the Feature extraction software were used to detect if probes were significantly detected above background (ie rejecting the null hypothesis that the probes are equal to the background signal). Only probes with a P-value <0.05 in 60% of the samples per donor group were included. Subsequently, probes with no entrezID annotation and duplicates were filtered out. Data was log2-transformed and quantile normalized [3].

Exact instructions are available in the accompanying ‘Additional_file_10.html’.

**Microbiota analysis**

Analysis of 454 pyrosequencing output was performed using *dada2* [4], optimized for pyrosequencing. This allows for the discrimination of amplicon sequence variants (ASVs), resolving differences in sequenced amplicons down to single nucleotides and replacing operational taxonomic unit (OTU) clustering thresholds. While this framework was developed for Illumina-based sequencing technology, analysis of pyrosequencing is supported. Specific parameter settings chosen for pyrosequencing include a HOMOPOLYMER_GAP_PENALTY=-1 and BAND_SIZE=32, with MAX_LEN=500 filtering applied.

Following creation of a count table of ASVs, taxonomic assignment was performed against the RDP data set (v16), specifically trained for use with *dada2*. A phylogenetic tree was created with the Phangorn package (v2.5.3) [5], using multiple sequence alignments generated by the DECIPHER package (v2.12.0) [6].

Further details are available in the accompanying R markdown file ‘Additional_file_9.html’. We follow closely he analysis pipeline recommended by the authors, using *dada2* and the *phyloseq* environment [7].

**Enterotype classification**

Data including 278 human samples from the Metahit consortia [8] was downloaded and enterotype classification performed as described by the original authors [9]. Following ‘harmonization’ of the two data sets (which involved matching genera in rows and adding zeroes to those genera in one dataset which were exclusive to the other), calculation of the Jensen-Shannon Distance (JSD) followed by Partitioning around Mediods (PAM) clustering to determine enterotypes. To avoid distorting the resulting PCoA plot significantly by the addition of a large volume of new samples, sample proportions were averaged at each time point and for each donor, resulting in 8 total representative samples submitted to enterotype classification. The samples were also submitted to reference-based enterotyping online, confirming that the four averaged samples from donor A belonged to the *Prevotella* enterotype, while the four averaged samples from donor B belonged to the *Bacteroides* enterotype. 7 out of 8 of these samples were defined as being within the ‘enterotype space’ by the reference-based enterotyping method (the averaged samples from donor A at time point 0 did not).

|  | BarcodeSequence |
| --- | --- |
| A11.D0 | ACGAGTGCGT |
| A12.D0 | ACGCTCGACA |
| A11.D11 | ATATCGCGAG |
| A11.D18 | AGCACTGTAG |
| A11.D8 | CTCGCGTGTC |
| A12.D11 | CGTGTCTCTA |
| A12.D18 | ATCAGACACG |
| A12.D8 | TAGTATCAGC |
| A21.D11 | CTCGCGTGTC |
| A21.D18 | ATATCGCGAG |
| A21.D8 | TCTCTATGCG |
| A22.D0 | AGACGCACTC |
| A22.D11 | TAGTATCAGC |
| A22.D18 | CGTGTCTCTA |
| A22.D8 | ACGAGTGCGT |
| B11.D11 | TCTCTATGCG |
| B11.D18 | CTCGCGTGTC |
| B11.D8 | ACGCTCGACA |
| B12.D0 | ATCAGACACG |
| B12.D11 | ACGAGTGCGT |
| B12.D18 | TAGTATCAGC |
| B12.D8 | AGACGCACTC |
| B21.D0 | ATATCGCGAG |
| B21.D11 | ACGCTCGACA |
| B21.D18 | TCTCTATGCG |
| B21.D8 | AGCACTGTAG |
| B22.D0 | CGTGTCTCTA |
| B22.D11 | AGACGCACTC |
| B22.D18 | TGATACGTCT |
| B22.D8 | ATCAGACACG |

Table: sequencing barcodes

**References**

1. R: **R: A language and environment for statistical computing.**: R Foundation for Statistical Computing, Vienna, Austria. URL <http://www.R-project.org/>; 2018.

2. Dray S, Dufour A-B: **The ade4 Package: Implementing the Duality Diagram for Ecologists.** *2007* 2007, **22:**20.

3. Bolstad BM, Irizarry RA, Astrand M, Speed TP: **A comparison of normalization methods for high density oligonucleotide array data based on variance and bias.** *Bioinformatics* 2003, **19:**185-193.

4. Callahan BJ, McMurdie PJ, Rosen MJ, Han AW, Johnson AJA, Holmes SP: **DADA2: High-resolution sample inference from Illumina amplicon data.** *Nature Methods* 2016, **13:**581.

5. Schliep KP: **phangorn: phylogenetic analysis in R.** *Bioinformatics* 2010, **27:**592-593.

6. Wright ES: **DECIPHER: harnessing local sequence context to improve protein multiple sequence alignment.** *BMC Bioinformatics* 2015, **16:**322.

7. Callahan BJ, Sankaran K, Fukuyama JA, McMurdie PJ, Holmes SP: **Bioconductor Workflow for Microbiome Data Analysis: from raw reads to community analyses.** *F1000Res* 2016, **5:**1492.

8. Le Chatelier E, Nielsen T, Qin J, Prifti E, Hildebrand F, Falony G, Almeida M, Arumugam M, Batto J-M, Kennedy S, et al: **Richness of human gut microbiome correlates with metabolic markers.** *Nature* 2013, **500:**541.

9. <http://enterotypes.org/>: **Accessed on 4 September 2018.**
