# Supplementary material for: Baseline microbiota composition modulates antibiotic-mediated effects on the gut microbiota and host
Source: Microbiome. 2019 Aug 2;7:111. doi: 10.1186/s40168-019-0725-3 (PMC6676565; doi:10.1186/s40168-019-0725-3)
Supplement: Supplementary file 9 — Output describing the code used for microbiota analysis. (HTML 3490 kb) [file 40168_2019_725_MOESM9_ESM.html]

Basal microbiota composition modulates antibiotic-mediated effects on the gut microbiota and host


# Basal microbiota composition modulates antibiotic-mediated effects on the gut microbiota and host

#### July 8, 2019

```
knitr::opts_chunk$set(message=FALSE, warning=FALSE, echo=TRUE)
```

## Amplicon analysis

Study examining effect of co-amoxiclav on the profiles of humanised gnotobiotic mice, humanised by two separate healthy donors.

This analysis follows that recommended in: “Callahan BJ, Sankaran K, Fukuyama JA, McMurdie PJ, Holmes SP: Bioconductor Workflow for Microbiome Data Analysis: from raw reads to community analyses. F1000Res 2016, 5:1492”

Some recommended parameter changes have been incorporated for pyrosequencing data.

Packages used here will need to be installed. Note that to exactly reproduce these figures, in addition to the set.seed() function, the same package version of dada2 (v1.10.0) will be required. See sessionInfo at the end for further details.

authors: “Aonghus Lavelle, Thomas Walter Hoffmann, Hang-Phuong Pham, Philippe Langella, Eric Guedon, Harry Sokol”

email: study(“harry.sokol@aphp.fr”); pipeline(“aonghuslavelle@gmail.com”)

part: “Part 1 - microbiota analysis”

```
library("knitr")
library("BiocStyle")
library("ggplot2")
library("gridExtra")
library("dada2")
library("phyloseq")
library("DECIPHER")
library("phangorn")
library("ggpubr")
library("RColorBrewer")
library("ggrepel")
set.seed(100)

#set path to directory with fastq files
data_directory_microbiota <- "/home/aonghus/Desktop/Final_data/Data_folder/Microbiota_data/fastq"
data_directory_microarray <- "/home/aonghus/Desktop/Final_data/Data_folder/Microarray_data"
setwd(data_directory_microbiota)
path <- data_directory_microbiota
list.files(path)
```

```
##  [1] "A11.co0.fastq.gz"         "A11.co11.fastq.gz"       
##  [3] "A11.co18.fastq.gz"        "A11.co8.fastq.gz"        
##  [5] "A12.co0.fastq.gz"         "A12.co11.fastq.gz"       
##  [7] "A12.co18.fastq.gz"        "A12.co8.fastq.gz"        
##  [9] "A21.co11.fastq.gz"        "A21.co18.fastq.gz"       
## [11] "A21.co8.fastq.gz"         "A22.co0.fastq.gz"        
## [13] "A22.co11.fastq.gz"        "A22.co18.fastq.gz"       
## [15] "A22.co8.fastq.gz"         "B11.co11.fastq.gz"       
## [17] "B11.co18.fastq.gz"        "B11.co8.fastq.gz"        
## [19] "B12.co0.fastq.gz"         "B12.co11.fastq.gz"       
## [21] "B12.co18.fastq.gz"        "B12.co8.fastq.gz"        
## [23] "B21.co0.fastq.gz"         "B21.co11.fastq.gz"       
## [25] "B21.co18.fastq.gz"        "B21.co8.fastq.gz"        
## [27] "B22.co0.fastq.gz"         "B22.co11.fastq.gz"       
## [29] "B22.co18.fastq.gz"        "B22.co8.fastq.gz"        
## [31] "filtered_use_qual_scores"
```

```
fnFs <- sort(list.files(path, pattern=".fastq.gz", full.names = TRUE))
sample.names <- sapply(strsplit(basename(fnFs), "_"), `[`, 1)
```

## Quality plots

Donor A

```
plotQualityProfile(fnFs[1:15])
```

Donor B

```
plotQualityProfile(fnFs[16:30])
```

```
#create filtered read directory
filt_path <- file.path(path, "filtered_use_qual_scores") 
filtFs <- file.path(filt_path, paste0(sample.names, "_filt.fastq.gz"))

#filter
out <- filterAndTrim(fnFs, filtFs, trimLeft=10, truncLen=350, maxN=0, maxEE=2, truncQ=2, maxLen=500, rm.phix=TRUE, compress=TRUE, multithread=FALSE)

#dereplicate
derepFs <- derepFastq(filtFs, verbose=TRUE)
sam.names <- sapply(strsplit(basename(filtFs), "_"), '[', 1)
names(derepFs) <- sam.names

#create table note additional pyrosequencing arguments
dadaFs <- dada(derepFs, err=NULL, multithread=FALSE, HOMOPOLYMER_GAP_PENALTY=-1, BAND_SIZE=32, selfConsist = TRUE, USE_QUALS=TRUE)
```

```
## Initializing error rates to maximum possible estimate.
## selfConsist step 1 ..............................
##    selfConsist step 2
##    selfConsist step 3
##    selfConsist step 4
##    selfConsist step 5
##    selfConsist step 6
##    selfConsist step 7
## Convergence after  7  rounds.
```

```
#pooled inference
dadaFs_pooled <- dada(derepFs, err=dadaFs[[1]]$err_out, multithread=TRUE, HOMOPOLYMER_GAP_PENALTY=-1, BAND_SIZE=32, selfConsist = TRUE, pool=TRUE, USE_QUALS=TRUE)
```

```
## selfConsist step 1 .
##    selfConsist step 2
##    selfConsist step 3
##    selfConsist step 4
##    selfConsist step 5
## Convergence after  5  rounds.
```

```
seqtab.combined <- makeSequenceTable(dadaFs_pooled)
dim(seqtab.combined)
```

```
## [1]  30 651
```

```
table(nchar(getSequences(seqtab.combined)))
```

```
## 
## 340 
## 651
```

```
#remove bimeras
seqtab <- removeBimeraDenovo(seqtab.combined, method="consensus", multithread=TRUE, verbose=TRUE)
```

```
#taxonomy is assigned using the RDP classifier https://zenodo.org/record/801828#.XOlYlyaxVuQ
ref_fasta <- "~/Documents/rdp_train_set_16.fa.gz"
ref_fasta_add <- "~/Documents/rdp_species_assignment_16.fa.gz"

taxtab <- assignTaxonomy(seqtab, refFasta = ref_fasta)
taxtab <- addSpecies(taxtab, refFasta = ref_fasta_add, allowMultiple = FALSE, verbose = FALSE)
colnames(taxtab) <- c("Kingdom", "Phylum", "Class", "Order", "Family", "Genus", "Species")
```

# Building phylogenetic tree

```
#MSA and phylogenetic tree
seqs <- getSequences(seqtab)
names(seqs) <- seqs # This propagates to the tip labels of the tree
alignment <- AlignSeqs(DNAStringSet(seqs), anchor=NA)
```

```
## Determining distance matrix based on shared 8-mers:
## ===========================================================================
## 
## Time difference of 1.78 secs
## 
## Clustering into groups by similarity:
## ===========================================================================
## 
## Time difference of 0.12 secs
## 
## Aligning Sequences:
## ===========================================================================
## 
## Time difference of 3.31 secs
## 
## Iteration 1 of 2:
## 
## Determining distance matrix based on alignment:
## ===========================================================================
## 
## Time difference of 0.15 secs
## 
## Reclustering into groups by similarity:
## ===========================================================================
## 
## Time difference of 0.16 secs
## 
## Realigning Sequences:
## ===========================================================================
## 
## Time difference of 2.3 secs
## 
## Iteration 2 of 2:
## 
## Determining distance matrix based on alignment:
## ===========================================================================
## 
## Time difference of 0.17 secs
## 
## Reclustering into groups by similarity:
## ===========================================================================
## 
## Time difference of 0.11 secs
## 
## Realigning Sequences:
## ===========================================================================
## 
## Time difference of 0.97 secs
```

```
phang.align <- phyDat(as(alignment, "matrix"), type="DNA")
dm <- dist.ml(phang.align)
treeNJ <- NJ(dm) # Note, tip order != sequence order
fit = pml(treeNJ, data=phang.align)

## negative edges length changed to 0!

fitGTR <- update(fit, k=4, inv=0.2)
fitGTR <- optim.pml(fitGTR, model="GTR", optInv=TRUE, optGamma=TRUE,
                    rearrangement = "stochastic", control = pml.control(trace = 0))
detach("package:phangorn", unload=TRUE)
```

## Reading in mapping data

```
#read in mapping file
map<-read.table("microbiota_metadata.txt", header=T)


map$Day = factor(map$Day, levels = c("0", "8", "11", "18"))
rownames(seqtab) <- sapply(strsplit(rownames(seqtab), ".f"), '[', 1)

#check
rownames(seqtab) %in% rownames(map)
```

```
##  [1] TRUE TRUE TRUE TRUE TRUE TRUE TRUE TRUE TRUE TRUE TRUE TRUE TRUE TRUE
## [15] TRUE TRUE TRUE TRUE TRUE TRUE TRUE TRUE TRUE TRUE TRUE TRUE TRUE TRUE
## [29] TRUE TRUE
```

```
map <- map[rownames(map) %in% rownames(seqtab),]
map <- map[match(rownames(seqtab), rownames(map)),]

#check
rownames(seqtab) == rownames(map)
```

```
##  [1] TRUE TRUE TRUE TRUE TRUE TRUE TRUE TRUE TRUE TRUE TRUE TRUE TRUE TRUE
## [15] TRUE TRUE TRUE TRUE TRUE TRUE TRUE TRUE TRUE TRUE TRUE TRUE TRUE TRUE
## [29] TRUE TRUE
```

## Create a phyloseq object

```
ps_new <- phyloseq(otu_table(seqtab, taxa_are_rows=FALSE), 
               sample_data(map), 
               tax_table(taxtab),
               phy_tree(fitGTR$tree))
```

# Filter data

Prevalence filtering

```
# Remove phyla that are NA
table(tax_table(ps_new)[, "Phylum"], exclude = NULL)
```

```
## 
##  Actinobacteria   Bacteroidetes      Firmicutes  Proteobacteria 
##               9             195             274              20 
## Verrucomicrobia            <NA> 
##               1               6
```

```
ps1 <- subset_taxa(ps_new, !is.na(Phylum) & !Phylum %in% c("", "uncharacterized"))

# Prevalence filter at 0.05 - in this example, removes reads present in only one mouse
prev0 = apply(X = otu_table(ps1),
              MARGIN = ifelse(taxa_are_rows(ps1), yes = 1, no = 2),
              FUN = function(x){sum(x > 0)})
prevdf = data.frame(Prevalence = prev0,
                    TotalAbundance = taxa_sums(ps1),
                    tax_table(ps1))
keepPhyla = table(prevdf$Phylum)[(table(prevdf$Phylum) > 0)]
prevdf1 = subset(prevdf, Phylum %in% names(keepPhyla))

# threshold
prevalenceThreshold = 0.05 * nsamples(ps1)
prevalenceThreshold
```

```
## [1] 1.5
```

```
# filter
ps2 = prune_taxa((prev0 > prevalenceThreshold), ps1)
ps2
```

```
## phyloseq-class experiment-level object
## otu_table()   OTU Table:         [ 454 taxa and 30 samples ]
## sample_data() Sample Data:       [ 30 samples by 9 sample variables ]
## tax_table()   Taxonomy Table:    [ 454 taxa by 7 taxonomic ranks ]
## phy_tree()    Phylogenetic Tree: [ 454 tips and 452 internal nodes ]
```

```
#save file to microarray directory for downtream correlation
saveRDS(ps2, file = paste(data_directory_microarray, "ps_final_filtered_microbiota.RData", sep = "/"))
```

# Microbiota analysis

```
ps2.prop <- transform_sample_counts(ps2, function(x) x/sum(x))
ps2.prop.A <- subset_samples(ps2.prop, sample_data(ps2.prop)$Donnor == "A")
ps2.prop.B <- subset_samples(ps2.prop, sample_data(ps2.prop)$Donnor == "B")
```

```
######alpha diversity#####
library(RColorBrewer)
library(ggpubr)

#data is rarefied for significance testing
set.seed(1234)
ps_rare <- rarefy_even_depth(ps2)
p <- plot_richness(ps_rare, x="Day", measure = "Simpson", color = "Donnor") + geom_boxplot(varwidth = TRUE) + facet_grid(~Donnor + Day, space = "free", scales = "free") + scale_color_discrete("Donor") + xlab("Day") + ylab("Simpson Diversity") + geom_point()

p2 <- p + geom_point() + 
  theme(strip.background = element_blank(), strip.text.x = element_blank(), axis.text.x=element_blank(), axis.ticks.x=element_blank()) + theme_classic() + theme(text = element_text(size=22), axis.text = element_text(size=16) ,legend.position = "bottom") + scale_color_brewer("Donor", palette = "Set1") #4 by 6
```

## Plot Simpson diversity

```
p2
```

## Genus level taxonomy barplots

```
main.otus <- names(sort(taxa_sums(ps2.prop), TRUE)[1:40])
ps.subs   <- prune_taxa(main.otus, ps2.prop)

taxa.barplots <- plot_bar(ps.subs, x="Sample", y="Abundance", fill="Genus") + facet_grid(~Donnor + Day, space = "fixed", scales = "free") + theme_classic() +
  theme(axis.title.x=element_blank(),
        axis.text.x=element_blank(),
        axis.ticks.x=element_blank(), text = element_text(size=25), legend.position = "bottom") + guides(fill=guide_legend(nrow=7,byrow=TRUE)) # 6 by 8
```

## Plot Taxonomy (Genus)

```
taxa.barplots
```

# Beta diversity

Principal coordinate analysis of the weighted unifrac distance

```
library(vegan)

set.seed(1234)
out.donor.wuf <- ordinate(ps2.prop, method = "MDS", distance = "wunifrac")
evals.donor.wuf <- out.donor.wuf$values$Eigenvalues

p.donor.wuf <- plot_ordination(ps2.prop, out.donor.wuf, shape = "Day", color = "Donnor") +
  theme_classic() +  stat_ellipse(aes(group=interaction(sample_data(ps2.prop)$Day, sample_data(ps2.prop)$Donnor)), alpha = 0.9) + ggtitle("Weighted Unifrac all samples") + theme(plot.title = element_text(hjust = 0.5)) + theme(text = element_text(size=18))

p.donor.wuf <- p.donor.wuf + geom_point(size=4) + guides(size = FALSE) + scale_color_brewer("Donor", palette = "Set1")

set.seed(1234)
a.dist <- phyloseq::distance(ps2.prop.A, method = "wunifrac")
set.seed(1234)
A.adonis <- adonis(a.dist ~ sample_data(ps2.prop.A)$Day, permutations = 99999)
R2.A <- A.adonis$aov.tab$R2[1]
P.A <- A.adonis$aov.tab$Pr[1]
set.seed(1234)
b.dist <- phyloseq::distance(ps2.prop.B, method = "wunifrac")
set.seed(1234)
B.adonis <- adonis(b.dist ~ sample_data(ps2.prop.B)$Day, permutations = 99999)
R2.B <- B.adonis$aov.tab$R2[1]
P.B <- B.adonis$aov.tab$Pr[1]

p.donor.wuf <- p.donor.wuf + annotate("text", size = 6, x = -0.4, y = 0.15, label = sprintf("italic(R)^2 == %s", round(A.adonis$aov.tab$R2[1],3)), parse = TRUE) + annotate("text", size = 6, x = -0.4, y = 0.05, label = sprintf("italic(P) == %s", round(A.adonis$aov.tab$Pr[1], 5)), parse = TRUE) + annotate("text", size = 6, x = 0.2, y = -0.25, label = sprintf("italic(R)^2 == %s", round(B.adonis$aov.tab$R2[1],3)), parse = TRUE) + annotate("text", size = 6, x = 0.2, y = -0.35, label = sprintf("italic(P) == %s", round(B.adonis$aov.tab$Pr[1], 5)), parse = TRUE) + annotate("rect", xmin = 0.15, ymin = -0.4, xmax = 0.25, ymax = -0.2, alpha = 0.2, fill = brewer.pal(4, "Set1")[2]) + annotate("rect", xmin = -0.45, ymin = 0.0, xmax = -0.35, ymax = 0.2, alpha = 0.2, fill = brewer.pal(4, "Set1")[1])
```

## Weighted Unifrac

```
p.donor.wuf
```

### Correlate genus abundance with PCoA axes 1 and 2 for donor A

```
#Donor A
ps.x <- ps2.prop.A

set.seed(1234)
out.donor.wuf <- ordinate(ps.x, method = "MDS", distance = "wunifrac")
evals.donor.wuf <- out.donor.wuf$values$Eigenvalues
p.donor.wuf <- plot_ordination(ps.x, out.donor.wuf, color = "Day", shape = "Day") + 
  theme_classic() +  stat_ellipse(aes(group=interaction(sample_data(ps.x)$Day, sample_data(ps.x)$Donnor)), alpha = 0.9) + ggtitle("Weighted unifrac donor A") + theme(plot.title = element_text(hjust = 0.5)) + theme(text = element_text(size=18))

p.donor.wuf <- p.donor.wuf + geom_point(size=4) + guides(size = FALSE) + scale_color_brewer("Day", palette = "Set2")


ps.x <- tax_glom(ps.x, "Genus")

biplot_otu_table_16S = as(t(otu_table(ps.x)), "matrix")
all(rownames(biplot_otu_table_16S) == rownames(tax_table(ps.x)))
```

```
## [1] TRUE
```

```
rownames(biplot_otu_table_16S) <- tax_table(ps.x)[,6]

biplot_cca_coord_16S = t(out.donor.wuf$vectors[,1:2])[,colnames(biplot_otu_table_16S)]

axis1.cor = apply(biplot_otu_table_16S, 1, function(x) cor.test(x, biplot_cca_coord_16S[1,], method = "spearman")$estimate)
axis2.cor = apply(biplot_otu_table_16S, 1, function(x) cor.test(x, biplot_cca_coord_16S[2,], method = "spearman")$estimate)
axis1.p = apply(biplot_otu_table_16S, 1, function(x) cor.test(x, biplot_cca_coord_16S[1,], method = "spearman")$p.value)
axis2.p = apply(biplot_otu_table_16S, 1, function(x) cor.test(x, biplot_cca_coord_16S[2,], method = "spearman")$p.value)
axis1.p = p.adjust(axis1.p, method = "fdr")
axis2.p = p.adjust(axis2.p, method = "fdr")
axis1.cor[axis1.p > 0.1] = 0 # zero insignificant correlations
axis2.cor[axis2.p > 0.1] = 0
axis1.cor = axis1.cor[names((sort(abs(axis1.cor), decreasing = TRUE)))] # order by absolute value
axis2.cor = axis2.cor[names((sort(abs(axis2.cor), decreasing = TRUE)))]


top_corr_otu = unique(c(names(axis1.cor)[1:25], names(axis2.cor)[1:25]))
top_corr_otu <- top_corr_otu[!(is.na(top_corr_otu))]
vectors_ax1_end = axis1.cor[top_corr_otu] 
vectors_ax2_end = axis2.cor[top_corr_otu]
axis1.p.top = axis1.p[top_corr_otu] 
axis2.p.top = axis2.p[top_corr_otu]
vectors_df = data.frame(top_corr_otu, vectors_ax1_end, vectors_ax2_end, axis1.p.top, axis2.p.top)
vectors_df$abundance = apply(biplot_otu_table_16S[top_corr_otu,], 1, sum)
vectors_df <- vectors_df[!(vectors_df$vectors_ax1_end == 0 & vectors_df$vectors_ax2_end == 0),]
vectors_df
```

```
##                                top_corr_otu vectors_ax1_end
## Faecalibacterium           Faecalibacterium      -0.8857143
## Dorea                                 Dorea      -0.7383560
## Prevotella                       Prevotella       0.6678571
## Barnesiella                     Barnesiella       0.6613050
## Oscillibacter                 Oscillibacter       0.6464286
## Bacteroides                     Bacteroides       0.0000000
## Phascolarctobacterium Phascolarctobacterium       0.0000000
## Clostridium_XlVb           Clostridium_XlVb       0.0000000
## Odoribacter                     Odoribacter       0.0000000
##                       vectors_ax2_end axis1.p.top axis2.p.top   abundance
## Faecalibacterium            0.0000000  0.00000000  0.33538954 3.731544482
## Dorea                       0.0000000  0.03756754  0.81986380 0.050853179
## Prevotella                  0.0000000  0.09148621  0.27130716 3.987263422
## Barnesiella                 0.0000000  0.09148621  0.33436462 0.074914258
## Oscillibacter               0.0000000  0.09992599  0.49974353 0.061845207
## Bacteroides                 0.7857143  0.31286410  0.02674038 2.589488623
## Phascolarctobacterium       0.7000000  0.91363849  0.05485985 0.143039268
## Clostridium_XlVb            0.7531837  0.84406713  0.02674038 0.013528753
## Odoribacter                 0.7205190  0.19943339  0.03666425 0.003382069
```

```
p.otu.corr <- p.donor.wuf + geom_segment(data = vectors_df, inherit.aes = FALSE, show.legend = FALSE, arrow = arrow(angle = 15, type = "closed"),
                        aes(x = 0, y = 0, xend = vectors_ax1_end/4, yend = vectors_ax2_end/4)) + geom_text_repel(data = vectors_df, inherit.aes = FALSE,  nudge_y = -0.05,
                                                                                                                           aes(vectors_ax1_end/4, vectors_ax2_end/4, label = top_corr_otu), size = 5) + theme(text = element_text(size=18), legend.position = "bottom")
p.otu.corr
```

### Perform two-way PERMANOVA and posthoc test

```
library(RVAideMemoire)
set.seed(1234)
all.dist <- phyloseq::distance(ps2.prop, method = "wunifrac")

set.seed(1234)
all.adonis <- adonis(all.dist ~ sample_data(ps2.prop)$Donnor*sample_data(ps2.prop)$Day, permutations = 99999)
```

# results of two way PERMANOVA

```
#results of two way PERMANOVA
all.adonis
```

```
## 
## Call:
## adonis(formula = all.dist ~ sample_data(ps2.prop)$Donnor * sample_data(ps2.prop)$Day,      permutations = 99999) 
## 
## Permutation: free
## Number of permutations: 99999
## 
## Terms added sequentially (first to last)
## 
##                                                        Df SumsOfSqs
## sample_data(ps2.prop)$Donnor                            1   0.49200
## sample_data(ps2.prop)$Day                               3   0.15060
## sample_data(ps2.prop)$Donnor:sample_data(ps2.prop)$Day  3   0.07075
## Residuals                                              22   0.19402
## Total                                                  29   0.90737
##                                                        MeanSqs F.Model
## sample_data(ps2.prop)$Donnor                           0.49200  55.787
## sample_data(ps2.prop)$Day                              0.05020   5.692
## sample_data(ps2.prop)$Donnor:sample_data(ps2.prop)$Day 0.02358   2.674
## Residuals                                              0.00882        
## Total                                                                 
##                                                             R2  Pr(>F)    
## sample_data(ps2.prop)$Donnor                           0.54222   1e-05 ***
## sample_data(ps2.prop)$Day                              0.16597 0.00057 ***
## sample_data(ps2.prop)$Donnor:sample_data(ps2.prop)$Day 0.07798 0.03096 *  
## Residuals                                              0.21383            
## Total                                                  1.00000            
## ---
## Signif. codes:  0 '***' 0.001 '**' 0.01 '*' 0.05 '.' 0.1 ' ' 1
```

```
set.seed(1234)
tw.perm <- pairwise.perm.manova(all.dist, fact = interaction(sample_data(ps2.prop)$Donnor, sample_data(ps2.prop)$Day), nperm = 99999)
tw.perm
```

```
## 
##  Pairwise comparisons using permutation MANOVAs on a distance matrix 
## 
## data:  all.dist by interaction(sample_data(ps2.prop)$Donnor, sample_data(ps2.prop)$Day)
## 99999 permutations 
## 
##      A.0   B.0   A.8   B.8   A.11  B.11  A.18 
## B.0  0.133 -     -     -     -     -     -    
## A.8  0.120 0.044 -     -     -     -     -    
## B.8  0.044 0.160 0.044 -     -     -     -    
## A.11 0.308 0.044 0.044 0.044 -     -     -    
## B.11 0.044 0.160 0.044 0.514 0.044 -     -    
## A.18 0.044 0.044 0.044 0.044 0.160 0.044 -    
## B.18 0.044 0.145 0.044 0.120 0.044 0.385 0.044
## 
## P value adjustment method: fdr
```

```
A.corr.p <- tw.perm$p.value[grepl("A", rownames(tw.perm$p.value)), grepl("A", colnames(tw.perm$p.value))]
B.corr.p <- tw.perm$p.value[grepl("B", rownames(tw.perm$p.value)), grepl("B", colnames(tw.perm$p.value))]
```

# matrix (lower triangle) of FDR-corrected p-values for pairwise tests

```
A.corr.p
```

```
##             A.0        A.8 A.11 A.18
## A.8  0.12000000         NA   NA   NA
## A.11 0.30769231 0.04444444   NA   NA
## A.18 0.04444444 0.04444444 0.16   NA
```

```
B.corr.p
```

```
##            B.0       B.8      B.11
## B.0         NA        NA        NA
## B.8  0.1600000        NA        NA
## B.11 0.1600000 0.5142857        NA
## B.18 0.1454545 0.1200000 0.3851852
```

### Correlate genus abundance with PCoA axes 1 and 2 for donor B

```
#Donor B
ps.x <- ps2.prop.B

set.seed(1234)
out.donor.wuf <- ordinate(ps.x, method = "MDS", distance = "wunifrac")
evals.donor.wuf <- out.donor.wuf$values$Eigenvalues
p.donor.wuf <- plot_ordination(ps.x, out.donor.wuf, color = "Day", shape = "Day") + 
  theme_classic() +  stat_ellipse(aes(group=interaction(sample_data(ps.x)$Day, sample_data(ps.x)$Donnor)), alpha = 0.9) + ggtitle("Weighted unifrac donor B") + theme(plot.title = element_text(hjust = 0.5)) + theme(text = element_text(size=18))

p.donor.wuf <- p.donor.wuf + geom_point(size=4) + guides(size = FALSE) + scale_color_brewer("Day", palette = "Set2")


ps.x <- tax_glom(ps.x, "Genus")

biplot_otu_table_16S = as(t(otu_table(ps.x)), "matrix")
all(rownames(biplot_otu_table_16S) == rownames(tax_table(ps.x)))
```

```
## [1] TRUE
```

```
rownames(biplot_otu_table_16S) <- tax_table(ps.x)[,6]

biplot_cca_coord_16S = t(out.donor.wuf$vectors[,1:2])[,colnames(biplot_otu_table_16S)]

axis1.cor = apply(biplot_otu_table_16S, 1, function(x) cor.test(x, biplot_cca_coord_16S[1,], method = "spearman")$estimate)
axis2.cor = apply(biplot_otu_table_16S, 1, function(x) cor.test(x, biplot_cca_coord_16S[2,], method = "spearman")$estimate)
axis1.p = apply(biplot_otu_table_16S, 1, function(x) cor.test(x, biplot_cca_coord_16S[1,], method = "spearman")$p.value)
axis2.p = apply(biplot_otu_table_16S, 1, function(x) cor.test(x, biplot_cca_coord_16S[2,], method = "spearman")$p.value)
axis1.p = p.adjust(axis1.p, method = "fdr")
axis2.p = p.adjust(axis2.p, method = "fdr")
axis1.cor[axis1.p > 0.1] = 0 # zero insignificant correlations
axis2.cor[axis2.p > 0.1] = 0
axis1.cor = axis1.cor[names((sort(abs(axis1.cor), decreasing = TRUE)))] # order by absolute value
axis2.cor = axis2.cor[names((sort(abs(axis2.cor), decreasing = TRUE)))]


top_corr_otu = unique(c(names(axis1.cor)[1:25], names(axis2.cor)[1:25]))
top_corr_otu <- top_corr_otu[!(is.na(top_corr_otu))]
vectors_ax1_end = axis1.cor[top_corr_otu] 
vectors_ax2_end = axis2.cor[top_corr_otu]
axis1.p.top = axis1.p[top_corr_otu] 
axis2.p.top = axis2.p[top_corr_otu]
vectors_df = data.frame(top_corr_otu, vectors_ax1_end, vectors_ax2_end, axis1.p.top, axis2.p.top)
vectors_df$abundance = apply(biplot_otu_table_16S[top_corr_otu,], 1, sum)
vectors_df <- vectors_df[!(vectors_df$vectors_ax1_end == 0 & vectors_df$vectors_ax2_end == 0),]
vectors_df
```

```
## [1] top_corr_otu    vectors_ax1_end vectors_ax2_end axis1.p.top    
## [5] axis2.p.top     abundance      
## <0 rows> (or 0-length row.names)
```

```
#no sig.

p.donor.wuf
```

## Differential abundance testing with DESeq2

Donor A

```
library(DESeq2)
library(ggpubr)
library(RColorBrewer)


ps.x <- tax_glom(ps2, "Genus")
ps.x <- subset_samples(ps.x, sample_data(ps2)$Donnor == "A")

var = colnames(sample_data(ps.x))[7] # insert number here
var1 = "Day" #####set variable here for plots if want to be different from var
tax.level <- "agglom";

# 2. Assign various variables to unique levels and number of levels
var.levels <- levels(unlist(sample_data(ps.x)[,var]))
n.levels <- length(var.levels)

# 3. Create empty list, counter and loop through all combinations, removing redundant ones
contrasts <- list()
counter <- 1
for (i in 1:n.levels){
  for (j in 1:n.levels){
    if (j > i){
      x <- c(i,j)
      contrasts[[counter]] <- x
      counter = counter + 1
    }
  }
}
n.cont <- length(contrasts)


# 4. Make a list to hold plots
DESeq2_plots <- vector("list", length = n.cont) # this will need to be renamed for each new variable
gm_mean = function(x, na.rm=TRUE){exp(sum(log(x[x > 0]),na.rm=na.rm)/length(x))} # Custom geometric mean

# 5. Make a colour vector to keep phylum colour assignments consistent between plots
dd <- unique(tax_table(ps.x)[,2])
dd.col <- brewer.pal(9, "Set1")[3:9]
names(dd.col)  <- dd

# 6. Perform initial DESeq set-up. NOTE: have to add variable manually
deseq2_day = phyloseq_to_deseq2(ps.x, ~ Day) #manually add this
deseq2_geoMeans = apply(counts(deseq2_day), 1, gm_mean)
deseq2_day = estimateSizeFactors(deseq2_day, type="poscounts", geoMeans = deseq2_geoMeans)
deseq2_day = DESeq(deseq2_day, test="Wald", fitType="parametric")

# 7. Select significance threshold
alpha.var <- 0.01


#create individual boxplots that are significant by Mann-Whitney
ps.x <- transform_sample_counts(ps.x, function(x) {x/sum(x)})


tax_table(ps.x) <- cbind(tax_table(ps.x), OTUs = tax_table(ps.x)[,6])
colnames(tax_table(ps.x))[ncol(tax_table(ps.x))] <- "OTUs"


pal <- brewer.pal(4, "Set2")

# 8. Loop through contrasts #change if using OTUs (102) or agglomerated genus (103/104)
genera <- c()
for (i in 1:n.cont){
  contrast.1 = var.levels[contrasts[[i]][1]]
  contrast.2 = var.levels[contrasts[[i]][2]]
  deseq2_day_X_vs_Y = results(deseq2_day, cooksCutoff = FALSE, contrast=c(var, contrast.1,contrast.2))
  #deseq2_day_X_vs_Y = cbind(as(deseq2_day_X_vs_Y, "data.frame"), Genus = otu.vec, OTUs = otu.vec, Phylum = phylum)
  deseq2_day_X_vs_Y = cbind(as(deseq2_day_X_vs_Y, "data.frame"),
                            as(tax_table(ps.x)[rownames(deseq2_day_X_vs_Y), ], "matrix"))
  
  deseq2_day_X_vs_Y_sigtab = deseq2_day_X_vs_Y[which(deseq2_day_X_vs_Y$padj < alpha.var), ]
  deseq2_day_X_vs_Y_sigtab = 
    deseq2_day_X_vs_Y_sigtab[order(deseq2_day_X_vs_Y_sigtab$log2FoldChange, decreasing = FALSE),]
  #deseq2_day_X_vs_Y_sigtab = deseq2_day_X_vs_Y_sigtab[!(deseq2_day_X_vs_Y_sigtab$Genus == "Escherichia/Shigella"),]
  deseq2_day_X_vs_Y_sigtab$Genus = factor(deseq2_day_X_vs_Y_sigtab$Genus,
                                          levels = unique(deseq2_day_X_vs_Y_sigtab$Genus)) # Order by fold-change switch to combined if OTUs
  genera <- c(genera, as.character(deseq2_day_X_vs_Y_sigtab$Genus))
   if(nrow(deseq2_day_X_vs_Y_sigtab) > 0){
    DESeq2_plots[[i]] <- ggplot(deseq2_day_X_vs_Y_sigtab, aes(y = Genus, x = log2FoldChange, colour = Phylum)) + geom_point(size=3) + theme_classic() + theme(text = element_text(size=22), legend.text = element_text(size=14)) + scale_color_manual(values = dd.col) +
      geom_vline(xintercept = 0, colour = "Red", lty = 2) + theme(legend.position="bottom") + ggtitle(paste(contrast.1, "versus", contrast.2, sep = " ")) + guides(colour=guide_legend(nrow = 2))

    plot(DESeq2_plots[[i]])
}
}
```

```
genera <- unique(genera)
otu.all.genera <- data.frame(Abundance = numeric(), Day = factor(), Genus = factor())
ps2.prop <- transform_sample_counts(ps2, function (x) {x/sum(x)})
ps2.genus.prop <- tax_glom(ps2.prop, "Genus")
#ps.x <- subset_samples(ps2.genus.prop, sample_data(ps2.genus.prop)$Donnor == "A")
for (i in 1:length(genera)){
  ps.genus <- subset_taxa(ps.x, Genus == genera[i])
  otu.genus <- otu_table(ps.genus)
  factors <- factor(sample_data(ps.genus)$Day, levels = c("0", "8", "11", "18"))
  genus.deseq2 <- rep(genera[i], nrow(otu.genus))
  otu.genus <- data.frame(Abundance = rowSums(otu.genus), Day = factors, Genus = genus.deseq2)
  otu.all.genera <- rbind(otu.all.genera, otu.genus)
}
my_comparisons <- list(c("0", "8"), c("0", "11"), c("0", "18"), c("8", "11"), c("8", "18"), c("11", "18"))
otu.all.genera$Abundance <- otu.all.genera$Abundance*100

ggboxplot(otu.all.genera, x="Day", y="Abundance", color = "Day", 
          outlier.shape = NA, palette = "Set2") + geom_jitter(aes(color = Day), size=1.6 ) + facet_wrap(~Genus, scales="free_y") + theme(text = element_text(size=22), legend.position = "bottom") + ggtitle("Donor A") + stat_compare_means(comparisons = my_comparisons, label = "p.signif")
```

Donor B

```
ps.x <- tax_glom(ps2, "Genus")
ps.x <- subset_samples(ps.x, sample_data(ps.x)$Donnor == "B")

var = colnames(sample_data(ps.x))[7] # insert number here
var1 = "Day" #####set variable here for plots if want to be different from var
tax.level <- "agglom";

# 2. Assign various variables to unique levels and number of levels
var.levels <- levels(unlist(sample_data(ps.x)[,var]))
n.levels <- length(var.levels)

# 3. Create empty list, counter and loop through all combinations, removing redundant ones
contrasts <- list()
counter <- 1
for (i in 1:n.levels){
  for (j in 1:n.levels){
    if (j > i){
      x <- c(i,j)
      contrasts[[counter]] <- x
      counter = counter + 1
    }
  }
}
n.cont <- length(contrasts)


# 4. Make a list to hold plots
DESeq2_plots <- vector("list", length = n.cont) # this will need to be renamed for each new variable
gm_mean = function(x, na.rm=TRUE){exp(sum(log(x[x > 0]),na.rm=na.rm)/length(x))} # Custom geometric mean

# 5. Make a colour vector to keep phylum colour assignments consistent between plots
dd <- unique(tax_table(ps.x)[,2])
dd.col <- c(brewer.pal(9, "Set1"), "#000000")
names(dd.col)  <- dd

# 6. Perform initial DESeq set-up. NOTE: have to add variable manually
deseq2_day = phyloseq_to_deseq2(ps.x, ~ Day) #manually add this
deseq2_geoMeans = apply(counts(deseq2_day), 1, gm_mean)
deseq2_day = estimateSizeFactors(deseq2_day, type="poscounts", geoMeans = deseq2_geoMeans)
deseq2_day = DESeq(deseq2_day, test="Wald", fitType="parametric")

# 7. Select significance threshold
alpha.var <- 0.01


#create individual boxplots that are significant by Mann-Whitney
ps.x <- transform_sample_counts(ps.x, function(x) {x/sum(x)})


tax_table(ps.x) <- cbind(tax_table(ps.x), OTUs = tax_table(ps.x)[,6])
colnames(tax_table(ps.x))[ncol(tax_table(ps.x))] <- "OTUs"


pal <- brewer.pal(4, "Set2")

# 8. Loop through contrasts #change if using OTUs (102) or agglomerated genus (103/104)
genera <- c()
for (i in 1:n.cont){
  contrast.1 = var.levels[contrasts[[i]][1]]
  contrast.2 = var.levels[contrasts[[i]][2]]
  deseq2_day_X_vs_Y = results(deseq2_day, cooksCutoff = FALSE, contrast=c(var, contrast.1,contrast.2))
  #deseq2_day_X_vs_Y = cbind(as(deseq2_day_X_vs_Y, "data.frame"), Genus = otu.vec, OTUs = otu.vec, Phylum = phylum)
  deseq2_day_X_vs_Y = cbind(as(deseq2_day_X_vs_Y, "data.frame"),
                            as(tax_table(ps.x)[rownames(deseq2_day_X_vs_Y), ], "matrix"))
  
  deseq2_day_X_vs_Y_sigtab = deseq2_day_X_vs_Y[which(deseq2_day_X_vs_Y$padj < alpha.var), ]
  deseq2_day_X_vs_Y_sigtab = 
    deseq2_day_X_vs_Y_sigtab[order(deseq2_day_X_vs_Y_sigtab$log2FoldChange, decreasing = FALSE),]
  deseq2_day_X_vs_Y_sigtab$Genus = factor(deseq2_day_X_vs_Y_sigtab$Genus,
                                          levels = unique(deseq2_day_X_vs_Y_sigtab$Genus)) # Order by fold-change switch to combined if OTUs
  genera <- c(genera, as.character(deseq2_day_X_vs_Y_sigtab$Genus))
  if(nrow(deseq2_day_X_vs_Y_sigtab) > 0){
    DESeq2_plots[[i]] <- ggplot(deseq2_day_X_vs_Y_sigtab, aes(y = Genus, x = log2FoldChange, colour = Phylum)) + geom_point(size=3) + theme_classic() + theme(text = element_text(size=22), legend.text = element_text(size=14)) + scale_color_manual(values = dd.col) +
      geom_vline(xintercept = 0, colour = "Red", lty = 2) + theme(legend.position="bottom") + ggtitle(paste(contrast.1, "versus", contrast.2, sep = " ")) + guides(colour=guide_legend(nrow = 2))
    
    plot(DESeq2_plots[[i]])
  }
}
```

```
genera <- unique(genera)
otu.all.genera <- data.frame(Abundance = numeric(), Day = factor(), Genus = factor())
ps2.prop <- transform_sample_counts(ps2, function (x) {x/sum(x)})
ps2.genus.prop <- tax_glom(ps2.prop, "Genus")
ps.x <- subset_samples(ps2.genus.prop, sample_data(ps2.genus.prop)$Donnor == "B")
for (i in 1:length(genera)){
  ps.genus <- subset_taxa(ps.x, Genus == genera[i])
  otu.genus <- otu_table(ps.genus)
  factors <- factor(sample_data(ps.genus)$Day, levels = c("0", "8", "11", "18"))
  genus.deseq2 <- rep(genera[i], nrow(otu.genus))
  otu.genus <- data.frame(Abundance = rowSums(otu.genus), Day = factors, Genus = genus.deseq2)
  otu.all.genera <- rbind(otu.all.genera, otu.genus)
}
my_comparisons <- list(c("0", "8"), c("0", "11"), c("0", "18"), c("8", "11"), c("8", "18"), c("11", "18"))
otu.all.genera$Abundance <- otu.all.genera$Abundance*100

ggboxplot(otu.all.genera, x="Day", y="Abundance", color = "Day", 
          outlier.shape = NA, palette = "Set2") + geom_jitter(aes(color = Day), size=1.6 ) + facet_wrap(~Genus, scales="free_y") + theme(text = element_text(size=22), legend.position = "bottom") + ggtitle("Donor B") + stat_compare_means(comparisons = my_comparisons, label = "p.signif")
```

# DESeq2 between donor groups

```
ps.x <- tax_glom(ps2, "Genus")

var = colnames(sample_data(ps.x))[4] # insert number here
var1 = "Donor" #####set variable here for plots if want to be different from var
tax.level <- "agglom";

# 2. Assign various variables to unique levels and number of levels
var.levels <- levels(unlist(sample_data(ps.x)[,var]))
n.levels <- length(var.levels)

# 3. Create empty list, counter and loop through all combinations, removing redundant ones
contrasts <- list()
counter <- 1
for (i in 1:n.levels){
  for (j in 1:n.levels){
    if (j > i){
      x <- c(i,j)
      contrasts[[counter]] <- x
      counter = counter + 1
    }
  }
}
n.cont <- length(contrasts)


# 4. Make a list to hold plots
DESeq2_plots <- vector("list", length = n.cont) # this will need to be renamed for each new variable
gm_mean = function(x, na.rm=TRUE){exp(sum(log(x[x > 0]),na.rm=na.rm)/length(x))} # Custom geometric mean

# 5. Make a colour vector to keep phylum colour assignments consistent between plots
dd <- unique(tax_table(ps.x)[,2])
dd.col <- brewer.pal(9, "Set1")[3:9]
names(dd.col)  <- dd

# 6. Perform initial DESeq set-up. NOTE: have to add variable manually
deseq2_day = phyloseq_to_deseq2(ps.x, ~ Donnor) #manually add this
deseq2_geoMeans = apply(counts(deseq2_day), 1, gm_mean)
deseq2_day = estimateSizeFactors(deseq2_day, type="poscounts", geoMeans = deseq2_geoMeans)
deseq2_day = DESeq(deseq2_day, test="Wald", fitType="parametric")

# 7. Select significance threshold
alpha.var <- 0.01


#create individual boxplots that are significant by Mann-Whitney
ps.x <- transform_sample_counts(ps.x, function(x) {x/sum(x)})


tax_table(ps.x) <- cbind(tax_table(ps.x), OTUs = tax_table(ps.x)[,6])
colnames(tax_table(ps.x))[ncol(tax_table(ps.x))] <- "OTUs"


pal <- brewer.pal(4, "Set2")

# 8. Loop through contrasts #change if using OTUs (102) or agglomerated genus (103/104)
genera <- c()
for (i in 1:n.cont){
  contrast.1 = var.levels[contrasts[[i]][1]]
  contrast.2 = var.levels[contrasts[[i]][2]]
  deseq2_day_X_vs_Y = results(deseq2_day, cooksCutoff = FALSE, contrast=c(var, contrast.1,contrast.2))
  #deseq2_day_X_vs_Y = cbind(as(deseq2_day_X_vs_Y, "data.frame"), Genus = otu.vec, OTUs = otu.vec, Phylum = phylum)
  deseq2_day_X_vs_Y = cbind(as(deseq2_day_X_vs_Y, "data.frame"),
                            as(tax_table(ps.x)[rownames(deseq2_day_X_vs_Y), ], "matrix"))
  
  deseq2_day_X_vs_Y_sigtab = deseq2_day_X_vs_Y[which(deseq2_day_X_vs_Y$padj < alpha.var), ]
  deseq2_day_X_vs_Y_sigtab = 
    deseq2_day_X_vs_Y_sigtab[order(deseq2_day_X_vs_Y_sigtab$log2FoldChange, decreasing = FALSE),]
  deseq2_day_X_vs_Y_sigtab$Genus = factor(deseq2_day_X_vs_Y_sigtab$Genus,
                                          levels = unique(deseq2_day_X_vs_Y_sigtab$Genus)) # Order by fold-change switch to combined if OTUs
  genera <- c(genera, as.character(deseq2_day_X_vs_Y_sigtab$Genus))
  if(nrow(deseq2_day_X_vs_Y_sigtab) > 0){
    DESeq2_plots[[i]] <- ggplot(deseq2_day_X_vs_Y_sigtab, aes(y = Genus, x = log2FoldChange, colour = Phylum)) + geom_point(size=3) + theme_classic() + theme(text = element_text(size=22), legend.text = element_text(size=14)) + scale_color_manual(values = dd.col) +
      geom_vline(xintercept = 0, colour = "Red", lty = 2) + theme(legend.position="bottom") + ggtitle(paste(contrast.1, "versus", contrast.2, sep = " ")) + guides(colour=guide_legend(nrow = 2))
    
    plot(DESeq2_plots[[i]])
  }
}
```

Distance-based analysis of antibiotic effects between cages

```
set.seed(1234)
all.dist <- phyloseq::distance(ps2.prop, method = "wunifrac")

Distance.df.A <- data.frame(Distance = numeric(), Comparison = character())
Distance.df.B <- data.frame(Distance = numeric(), Comparison = character())

set.seed(1234)
all.dist <- phyloseq::distance(ps2.prop, method = "wunifrac")
all.dist <-  as.matrix(all.dist)
A.dist <- all.dist[grepl("A", rownames(all.dist)), grepl("A", colnames(all.dist))]
B.dist <- all.dist[grepl("B", rownames(all.dist)), grepl("B", colnames(all.dist))]
if(all(rownames(A.dist) == rownames(sample_data(ps2.prop.A))) & all(colnames(A.dist) == rownames(sample_data(ps2.prop.A)))){
    #calculate distances between all timepoints in same cage (ie baseline fluctuation)
    A.dist.cage.1 <- A.dist[sample_data(ps2.prop.A)$Cage == 1, sample_data(ps2.prop.A)$Cage == 1]
    A.dist.cage.2 <- A.dist[sample_data(ps2.prop.A)$Cage == 2, sample_data(ps2.prop.A)$Cage == 2]
    Day.vec.1 <- unlist(lapply(rownames(A.dist.cage.1), function(x) strsplit(x, ".co")[[1]][2]))
    Day.vec.2 <- unlist(lapply(rownames(A.dist.cage.2), function(x) strsplit(x, ".co")[[1]][2]))
    D0.cage1 <- as.vector(A.dist.cage.1[Day.vec.1 != "0", Day.vec.1 == "0"])
    D8.cage1 <- as.vector(A.dist.cage.1[Day.vec.1 != "8", Day.vec.1 == "8"])
    D11.cage1 <- as.vector(A.dist.cage.1[Day.vec.1 != "11", Day.vec.1 == "11"])
    D18.cage1 <- as.vector(A.dist.cage.1[Day.vec.1 != "18", Day.vec.1 == "18"])
    D0.cage2 <- as.vector(A.dist.cage.2[Day.vec.2!= "0", Day.vec.2 == "0"])
    D8.cage2 <- as.vector(A.dist.cage.2[Day.vec.2 != "8", Day.vec.2 == "8"])
    D11.cage2 <- as.vector(A.dist.cage.2[Day.vec.2 != "11", Day.vec.2 == "11"])
    D18.cage2 <- as.vector(A.dist.cage.2[Day.vec.2 != "18", Day.vec.2 == "18"])
    Cage_distances.A <- c(D0.cage1, D8.cage1, D11.cage1, D18.cage1, D0.cage2, D8.cage2, D11.cage2, D18.cage2)
    Cage_distances.A <- unique(Cage_distances.A)
    #calculate distances between same timepoint in different cages
    D0 <- A.dist[grepl(".co0", rownames(A.dist)), grepl(".co0", colnames(A.dist))]
    diff.cages.0 <- as.vector(D0[lower.tri(D0)])
    D8 <- A.dist[grepl(".co8", rownames(A.dist)), grepl(".co8", colnames(A.dist))]
    diff.cages.8 <- as.vector(D8[lower.tri(D8)])
    D11 <- A.dist[grepl(".co11", rownames(A.dist)), grepl(".co11", colnames(A.dist))]
    diff.cages.11 <- as.vector(D11[lower.tri(D11)])
    D18 <- A.dist[grepl(".co18", rownames(A.dist)), grepl(".co18", colnames(A.dist))]
    diff.cages.18 <- as.vector(D18[lower.tri(D18)])
    #create a dataframe
    Distance.df.A <- rbind(Distance.df.A, data.frame(Distance = Cage_distances.A, Comparison = rep("Cage", length(Cage_distances.A))))
    Distance.df.A <- rbind(Distance.df.A, data.frame(Distance = diff.cages.0, Comparison = rep("D0", length(diff.cages.0))))
    Distance.df.A <- rbind(Distance.df.A, data.frame(Distance = diff.cages.8, Comparison = rep("D8", length(diff.cages.8))))
    Distance.df.A <- rbind(Distance.df.A, data.frame(Distance = diff.cages.11, Comparison = rep("D11", length(diff.cages.11))))
    Distance.df.A <- rbind(Distance.df.A, data.frame(Distance = diff.cages.18, Comparison = rep("D18", length(diff.cages.18))))
  }
  if(all(rownames(B.dist) == rownames(sample_data(ps2.prop.B))) & all(colnames(B.dist) == rownames(sample_data(ps2.prop.B)))){
    #calculate distances between all timepoints in same cage (ie baseline fluctuation)
    B.dist.cage.1 <- B.dist[sample_data(ps2.prop.B)$Cage == 1, sample_data(ps2.prop.B)$Cage == 1]
    B.dist.cage.2 <- B.dist[sample_data(ps2.prop.B)$Cage == 2, sample_data(ps2.prop.B)$Cage == 2]
    Day.vec.1 <- unlist(lapply(rownames(B.dist.cage.1), function(x) strsplit(x, ".co")[[1]][2]))
    Day.vec.2 <- unlist(lapply(rownames(B.dist.cage.2), function(x) strsplit(x, ".co")[[1]][2]))
    D0.cage1 <- as.vector(B.dist.cage.1[Day.vec.1 != "0", Day.vec.1 == "0"])
    D8.cage1 <- as.vector(B.dist.cage.1[Day.vec.1 != "8", Day.vec.1 == "8"])
    D11.cage1 <- as.vector(B.dist.cage.1[Day.vec.1 != "11", Day.vec.1 == "11"])
    D18.cage1 <- as.vector(B.dist.cage.1[Day.vec.1 != "18", Day.vec.1 == "18"])
    D0.cage2 <- as.vector(B.dist.cage.2[Day.vec.2!= "0", Day.vec.2 == "0"])
    D8.cage2 <- as.vector(B.dist.cage.2[Day.vec.2 != "8", Day.vec.2 == "8"])
    D11.cage2 <- as.vector(B.dist.cage.2[Day.vec.2 != "11", Day.vec.2 == "11"])
    D18.cage2 <- as.vector(B.dist.cage.2[Day.vec.2 != "18", Day.vec.2 == "18"])
    Cage_distances.B <- c(D0.cage1, D8.cage1, D11.cage1, D18.cage1, D0.cage2, D8.cage2, D11.cage2, D18.cage2)
    Cage_distances.B <- unique(Cage_distances.B)
    #calculate distances between same timepoint in different cages
    D0 <- B.dist[grepl(".co0", rownames(B.dist)), grepl(".co0", colnames(B.dist))]
    diff.cages.0 <- as.vector(D0[lower.tri(D0)])
    D8 <- B.dist[grepl(".co8", rownames(B.dist)), grepl(".co8", colnames(B.dist))]
    diff.cages.8 <- as.vector(D8[lower.tri(D8)])
    D11 <- B.dist[grepl(".co11", rownames(B.dist)), grepl(".co11", colnames(B.dist))]
    diff.cages.11 <- as.vector(D11[lower.tri(D11)])
    D18 <- B.dist[grepl(".co18", rownames(B.dist)), grepl(".co18", colnames(B.dist))]
    diff.cages.18 <- as.vector(D18[lower.tri(D18)])
    #create a dataframe
    Distance.df.B <- rbind(Distance.df.B, data.frame(Distance = Cage_distances.B, Comparison = rep("Cage", length(Cage_distances.B))))
    Distance.df.B <- rbind(Distance.df.B, data.frame(Distance = diff.cages.0, Comparison = rep("D0", length(diff.cages.0))))
    Distance.df.B <- rbind(Distance.df.B, data.frame(Distance = diff.cages.8, Comparison = rep("D8", length(diff.cages.8))))
    Distance.df.B <- rbind(Distance.df.B, data.frame(Distance = diff.cages.11, Comparison = rep("D11", length(diff.cages.11))))
    Distance.df.B <- rbind(Distance.df.B, data.frame(Distance = diff.cages.18, Comparison = rep("D18", length(diff.cages.18))))
}


Distance.df.A$Donor <- rep("A", nrow(Distance.df.A))
Distance.df.B$Donor <- rep("B", nrow(Distance.df.B))

Distance.df <- rbind(Distance.df.A, Distance.df.B)

my_comparisons <- list(c("Cage", "D8"))
ggplot(Distance.df, aes(Comparison, Distance, colour=Comparison)) + geom_boxplot() + geom_jitter() + facet_grid(~Donor) + stat_compare_means(comparisons = my_comparisons) +
  scale_colour_manual(values = c("black", brewer.pal(4, "Set2"))) + theme_classic()
```

# Session info

```
sessionInfo()
```

```
## R version 3.6.0 (2019-04-26)
## Platform: x86_64-pc-linux-gnu (64-bit)
## Running under: Ubuntu 18.04.2 LTS
## 
## Matrix products: default
## BLAS:   /usr/lib/x86_64-linux-gnu/blas/libblas.so.3.7.1
## LAPACK: /usr/lib/x86_64-linux-gnu/lapack/liblapack.so.3.7.1
## 
## locale:
##  [1] LC_CTYPE=en_US.UTF-8       LC_NUMERIC=C              
##  [3] LC_TIME=fr_FR.UTF-8        LC_COLLATE=en_US.UTF-8    
##  [5] LC_MONETARY=fr_FR.UTF-8    LC_MESSAGES=en_US.UTF-8   
##  [7] LC_PAPER=fr_FR.UTF-8       LC_NAME=C                 
##  [9] LC_ADDRESS=C               LC_TELEPHONE=C            
## [11] LC_MEASUREMENT=fr_FR.UTF-8 LC_IDENTIFICATION=C       
## 
## attached base packages:
## [1] stats4    parallel  stats     graphics  grDevices utils     datasets 
## [8] methods   base     
## 
## other attached packages:
##  [1] DESeq2_1.24.0               SummarizedExperiment_1.14.0
##  [3] DelayedArray_0.8.0          BiocParallel_1.16.6        
##  [5] matrixStats_0.54.0          Biobase_2.42.0             
##  [7] GenomicRanges_1.36.0        GenomeInfoDb_1.18.2        
##  [9] RVAideMemoire_0.9-73        vegan_2.5-5                
## [11] lattice_0.20-38             permute_0.9-5              
## [13] ggrepel_0.8.1               RColorBrewer_1.1-2         
## [15] ggpubr_0.2                  magrittr_1.5               
## [17] ape_5.3                     DECIPHER_2.12.0            
## [19] RSQLite_2.1.1               Biostrings_2.52.0          
## [21] XVector_0.24.0              IRanges_2.18.0             
## [23] S4Vectors_0.22.0            BiocGenerics_0.30.0        
## [25] phyloseq_1.24.2             dada2_1.10.0               
## [27] Rcpp_1.0.1                  gridExtra_2.3              
## [29] ggplot2_3.1.1               BiocStyle_2.12.0           
## [31] knitr_1.22                 
## 
## loaded via a namespace (and not attached):
##  [1] colorspace_1.4-1         ggsignif_0.5.0          
##  [3] hwriter_1.3.2            htmlTable_1.13.1        
##  [5] base64enc_0.1-3          rstudioapi_0.10         
##  [7] bit64_0.9-7              AnnotationDbi_1.46.0    
##  [9] codetools_0.2-16         splines_3.6.0           
## [11] geneplotter_1.58.0       ade4_1.7-13             
## [13] Formula_1.2-3            jsonlite_1.6            
## [15] Rsamtools_2.0.0          annotate_1.58.0         
## [17] cluster_2.0.8            BiocManager_1.30.4      
## [19] compiler_3.6.0           backports_1.1.4         
## [21] assertthat_0.2.1         Matrix_1.2-17           
## [23] lazyeval_0.2.2           acepack_1.4.1           
## [25] htmltools_0.3.6          tools_3.6.0             
## [27] igraph_1.2.4.1           gtable_0.3.0            
## [29] glue_1.3.1               GenomeInfoDbData_1.2.0  
## [31] reshape2_1.4.3           dplyr_0.8.0.1           
## [33] ShortRead_1.42.0         fastmatch_1.1-0         
## [35] multtest_2.36.0          nlme_3.1-139            
## [37] iterators_1.0.10         xfun_0.6                
## [39] stringr_1.4.0            XML_3.98-1.19           
## [41] zlibbioc_1.28.0          MASS_7.3-51.1           
## [43] scales_1.0.0             biomformat_1.12.0       
## [45] rhdf5_2.24.0             yaml_2.2.0              
## [47] memoise_1.1.0            rpart_4.1-15            
## [49] latticeExtra_0.6-28      stringi_1.4.3           
## [51] genefilter_1.62.0        foreach_1.4.4           
## [53] checkmate_1.9.3          rlang_0.3.4             
## [55] pkgconfig_2.0.2          bitops_1.0-6            
## [57] evaluate_0.13            purrr_0.3.2             
## [59] Rhdf5lib_1.2.1           GenomicAlignments_1.20.0
## [61] htmlwidgets_1.3          labeling_0.3            
## [63] bit_1.1-14               tidyselect_0.2.5        
## [65] plyr_1.8.4               R6_2.4.0                
## [67] Hmisc_4.2-0              DBI_1.0.0               
## [69] pillar_1.4.0             foreign_0.8-70          
## [71] withr_2.1.2              mgcv_1.8-28             
## [73] survival_2.43-3          RCurl_1.95-4.12         
## [75] nnet_7.3-12              tibble_2.1.1            
## [77] crayon_1.3.4             rmarkdown_1.12          
## [79] locfit_1.5-9.1           grid_3.6.0              
## [81] data.table_1.12.2        blob_1.1.1              
## [83] digest_0.6.18            xtable_1.8-4            
## [85] RcppParallel_4.4.2       munsell_0.5.0           
## [87] quadprog_1.5-7
```
