# Supplementary material for: Baseline microbiota composition modulates antibiotic-mediated effects on the gut microbiota and host
Source: Microbiome. 2019 Aug 2;7:111. doi: 10.1186/s40168-019-0725-3 (PMC6676565; doi:10.1186/s40168-019-0725-3)
Supplement: Supplementary file 10 — Output describing the code used for transcriptome analysis. (HTML 2420 kb) [file 40168_2019_725_MOESM10_ESM.html]

Basal microbiota composition modulates antibiotic-mediated effects on the gut microbiota and host


# Basal microbiota composition modulates antibiotic-mediated effects on the gut microbiota and host

#### July 8, 2019

```
knitr::opts_chunk$set(message=FALSE, warning=FALSE, echo=TRUE)
```

## Transcriptome analysis

Study examining effect of co-amoxiclav on the profiles of humanised gnotobiotic mice, humanised by two separate healthy donors.

authors: “Aonghus Lavelle, Thomas Walter Hoffmann, Hang-Phuong Pham, Philippe Langella, Eric Guedon, Harry Sokol”

email: study(“harry.sokol@aphp.fr”); pipeline(“aonghuslavelle@gmail.com”) part: “Part 2 - microarray analysis”

# Import data, filter based on detection, annotation, log2 transform and quantile normalize

Filtering steps: 1. Remove probes not detected

```
library("limma")
library("knitr")
library("statmod")
library("org.Mm.eg.db")
library("GSEABase")
library("GSVA")
library("Biobase")
library("genefilter")
library("snow")
library("preprocessCore")
library("ggplot2")
library("ade4")
library("factoextra")
library("GO.db")

data_directory_microarray <- "/home/aonghus/Desktop/Final_data/Data_folder/Microarray_data/"
setwd(data_directory_microarray)

#annotation file from Biomart 28/03/2019
entrezid <- read.table("Biomart_all_28032019.txt", na.strings = c("", "NA"), header = T, sep = "\t")

#remove probes without annotations
entrezid <- entrezid[!(is.na(entrezid$NCBI.gene.ID)),]
entrezid <- entrezid[order(entrezid$NCBI.gene.ID),]

#maps for raw files
detect_map <- read.table("microarray_metadata.txt", row.names = 1, header=T)
detect_map$Day <- factor(detect_map$Day, levels = c(0, 8, 11, 18))

#import raw and detect files
yfilt.detect <- read.table("gDetect_microarray.txt", row.names = 1, header=T)
colnames(yfilt.detect) <- gsub("gDetect_", "", colnames(yfilt.detect))
yfilt.raw <- read.table("gRaw_microarray.txt", row.names = 1, header=T)
colnames(yfilt.raw) <- gsub("gRaw_", "", colnames(yfilt.raw))

#order
detect_map <- detect_map[match(colnames(yfilt.detect), detect_map$Raw_Array_ID),]
all(colnames(yfilt.detect) == detect_map$Raw_Array_ID)
```

```
## [1] TRUE
```

```
all(colnames(yfilt.raw) == detect_map$Raw_Array_ID)
```

```
## [1] TRUE
```

```
#check
all(colnames(yfilt.detect) == detect_map$Raw_Array_ID)
```

```
## [1] TRUE
```

```
all(colnames(yfilt.raw) == detect_map$Raw_Array_ID)
```

```
## [1] TRUE
```

```
detect_map$S_Name <- gsub("-J", ".D", detect_map$S_Name)

colnames(yfilt.detect) <- detect_map$S_Name
colnames(yfilt.raw) <- detect_map$S_Name

#check
all(detect_map$S_Name == colnames(yfilt.detect))
```

```
## [1] TRUE
```

```
all(detect_map$S_Name == colnames(yfilt.raw))
```

```
## [1] TRUE
```

```
#filter only those with 60% probes in at least one group
len.A <- length(detect_map$A_Group[detect_map$Donor == "A"])*0.6
len.B <- length(detect_map$A_Group[detect_map$Donor == "B"])*0.6

#select probes
yfilt.raw <- yfilt.raw[rowSums(yfilt.detect[,detect_map$Donor == "A"] < 0.05) >= len.A | rowSums(yfilt.detect[,detect_map$Donor == "B"] < 0.05) >= len.B,]
yfilt.raw <- yfilt.raw[rownames(yfilt.raw) %in% entrezid$AGILENT.SurePrint.G3.GE.8x60k.probe,]

entrezid <- entrezid[entrezid$AGILENT.SurePrint.G3.GE.8x60k.probe %in% rownames(yfilt.raw),]
entrezid <- entrezid[order(entrezid$AGILENT.SurePrint.G3.GE.8x60k.probe),]

#This loop creates a logical vector to remove redundant probe IDs (column 4) in a sorted data frame
log.vec <- c(TRUE)
for (i in 2:nrow(entrezid)){
  if (entrezid[i,4] == entrezid[i-1,4]){
    log.vec <- c(log.vec, FALSE)
  } else {
    log.vec <- c(log.vec, TRUE)
  }
}
entrezid <- entrezid[log.vec,]

#remove redundancy in entrezIDs (column 3)
entrezid <- entrezid[order(entrezid$NCBI.gene.ID),]
log.vec <- c(TRUE)
for (i in 2:nrow(entrezid)){
  if (entrezid[i,3] == entrezid[i-1,3]){
    log.vec <- c(log.vec, FALSE)
  } else {
    log.vec <- c(log.vec, TRUE)
  }
}
entrezid <- entrezid[log.vec,]

#filter expression data to include unique entrezIDs
yfilt.raw <- yfilt.raw[rownames(yfilt.raw) %in% entrezid$AGILENT.SurePrint.G3.GE.8x60k.probe,]
#reorder annotation data frame to match expression data
entrezid <- entrezid[match(rownames(yfilt.raw), entrezid$AGILENT.SurePrint.G3.GE.8x60k.probe),]
#check
all(entrezid$AGILENT.SurePrint.G3.GE.8x60k.probe == rownames(yfilt.raw))
```

```
## [1] TRUE
```

```
#rename genes by entrezID
rownames(yfilt.raw) <- entrezid$NCBI.gene.ID

#log2-transform to stabilise variance
yfilt.transf <- log2(yfilt.raw)

#quantile normalise
yfilt.transf <- normalize.quantiles(as.matrix(yfilt.transf))
colnames(yfilt.transf) <- colnames(yfilt.raw)
rownames(yfilt.transf) <- rownames(yfilt.raw)

#rename to final annotated working data
yfilt.annot <- yfilt.transf

#make limited metadata
AB_variables <- data.frame(Donor = detect_map$Donor, Day = detect_map$Day)
rownames(AB_variables) <- detect_map$S_Name
AB_variables.adf <- as(AB_variables, "AnnotatedDataFrame")

AB_variables.A <- AB_variables[AB_variables$Donor == "A",]
AB_variables.B <- AB_variables[AB_variables$Donor == "B",]


rm(yfilt.raw, yfilt.detect, yfilt.transf, log.vec)
```

## PCA plots

```
library(ade4)
library(factoextra)
#all
gene.pac <- dudi.pca(t(yfilt.annot), nf=3, scannf = FALSE)
scrs <- gene.pac$li
scrs <- cbind(as.data.frame(scrs), AB_variables)
cent <-  aggregate(cbind(Axis1, Axis2) ~ Day, data=scrs, FUN=mean)
segs <- merge(scrs, setNames(cent, c("Day", "oAxis1", "oAxis2")), by = "Day", sort = FALSE)
dAll <- fviz_pca_ind(gene.pac, habillage = factor(AB_variables$Day, levels = c("0", "8", "11", "18")), addEllipses = TRUE, ellipse.level=0.68, geom = "point", label = "var") + scale_colour_manual("Day", values = c("#66C2A5", "#FC8D62", "#8DA0CB", "#E78AC3")) + theme_minimal() + ggtitle("PCA - All gene expression") + guides(fill=guide_legend(title = "Day"), shape=guide_legend(title = "Day"), color=guide_legend(title = "Day")) + geom_segment(data = segs, mapping = aes(x = Axis1, y = Axis2, xend = oAxis1, yend = oAxis2, color = Day)) + ylim(-230, 150) + xlim(-150, 190)

scrs <- gene.pac$li[AB_variables$Donor == "A",]
scrs <- cbind(as.data.frame(scrs), AB_variables.A)
cent <-  aggregate(cbind(Axis1, Axis2) ~ Day, data=scrs, FUN=mean)
segs <- merge(scrs, setNames(cent, c("Day", "oAxis1", "oAxis2")), by = "Day", sort = FALSE)
x <- rownames(gene.pac$li)[AB_variables$Donor == "A"]
dA <- fviz_pca_ind(gene.pac, habillage = factor(interaction(AB_variables$Donor, AB_variables$Day), levels = c("A.0", "A.8", "A.11", "A.18")), addEllipses = TRUE, ellipse.level=0.68, geom = "point", select.ind = list(name = x)) + scale_colour_manual(interaction(AB_variables$Donor, AB_variables$Day), values = c("#66C2A5", "#8DA0CB", "#E78AC3", "#FC8D62", "#66C2A5", "#8DA0CB", "#E78AC3", "#FC8D62")) + theme_minimal() + ggtitle("PCA - Donor A gene expression") + guides(fill=guide_legend(title = "Day"), shape=guide_legend(title = "Day")) + geom_segment(data = segs[segs$Donor == "A",], mapping = aes(x = Axis1, y = Axis2, xend = oAxis1, yend = oAxis2, color = Day)) + ylim(-230, 150) + xlim(-150, 190)


scrs <- gene.pac$li[AB_variables$Donor == "B",]
scrs <- cbind(as.data.frame(scrs), AB_variables.B)
cent <-  aggregate(cbind(Axis1, Axis2) ~ Day, data=scrs, FUN=mean)
segs <- merge(scrs, setNames(cent, c("Day", "oAxis1", "oAxis2")), by = "Day", sort = FALSE)
y <- rownames(gene.pac$li)[AB_variables$Donor == "B"]
dB <- fviz_pca_ind(gene.pac, habillage = factor(interaction(AB_variables$Donor, AB_variables$Day), levels = c("B.0", "B.8", "B.11", "B.18")), addEllipses = TRUE, ellipse.level=0.68, geom = "point", select.ind = list(name = y)) + scale_colour_manual(interaction(AB_variables$Donor, AB_variables$Day), values = c("#66C2A5", "#8DA0CB", "#E78AC3", "#FC8D62", "#66C2A5", "#8DA0CB", "#E78AC3", "#FC8D62")) + theme_minimal() + ggtitle("PCA - Donor B gene expression") + guides(fill=guide_legend(title = "Day"), shape=guide_legend(title = "Day"), color=guide_legend(title = "Day")) + geom_segment(data = segs, mapping = aes(x = Axis1, y = Axis2, xend = oAxis1, yend = oAxis2, color = Day)) + ylim(-230, 150) + xlim(-150, 190)
```

# Plot PCA plots

```
dAll
```

```
dA
```

```
dB
```

# Differential gene analysis with limma

Firstly for all mice combined

```
all(colnames(yfilt.annot) == rownames(AB_variables))
```

```
## [1] TRUE
```

```
donor.day.f <- factor(interaction("d",AB_variables$Day)) #subset by donor

lev.donor.day <- c("d.0", "d.8", "d.11", "d.18")

ddf <- factor(donor.day.f, levels=lev.donor.day)
donor.day.design.mat <- model.matrix(~0+ddf)
colnames(donor.day.design.mat) <- lev.donor.day

#check!

all(rownames(AB_variables) == colnames(yfilt.annot))
```

```
## [1] TRUE
```

```
cont.dd.mat <- makeContrasts(
  com.postAB = "d.8-d.0",
  com.rec1 = "d.11-d.8",
  com.rec1a = "d.11-d.0",
  com.rec2 = "d.18-d.8",
  com.rec2a = "d.18-d.0",
  com.rec3 = "d.18-d.11",
  levels=donor.day.design.mat)
dd.fit <- lmFit(yfilt.annot, donor.day.design.mat)
dd.fit2 <- contrasts.fit(dd.fit, cont.dd.mat)
dd.fit3 <- eBayes(dd.fit2)
dd.fit3$genes$EntrezID <- rownames(dd.fit3)

deg.postAB <- topTable(dd.fit3, coef="com.postAB", adjust="BH", p.value = 0.05, number=Inf)
deg.rec1 <- topTable(dd.fit3, coef="com.rec1", adjust="BH", p.value = 0.05, number=Inf)
deg.rec1a <- topTable(dd.fit3, coef="com.rec1a", adjust="BH", p.value = 0.05, number=Inf)
deg.rec2 <- topTable(dd.fit3, coef="com.rec2", adjust="BH", p.value = 0.05, number=Inf)
deg.rec2a <- topTable(dd.fit3, coef="com.rec2a", adjust="BH", p.value = 0.05, number=Inf)
deg.rec3 <- topTable(dd.fit3, coef="com.rec3", adjust="BH", p.value = 0.05, number=Inf)
dim(deg.postAB)[1]
```

```
## [1] 73
```

```
dim(deg.rec1)[1]
```

```
## [1] 624
```

```
dim(deg.rec1a)[1]
```

```
## [1] 2776
```

```
dim(deg.rec2)[1]
```

```
## [1] 459
```

```
dim(deg.rec2a)[1]
```

```
## [1] 1845
```

```
dim(deg.rec3)[1]
```

```
## [1] 0
```

```
GO.postAB <- goana(dd.fit3, coef="com.postAB", FDR = 0.05, species="Mm", geneid="EntrezID")
GO.rec1 <- goana(dd.fit3, coef="com.rec1", FDR = 0.05, species="Mm", geneid="EntrezID")
GO.rec1a <- goana(dd.fit3, coef="com.rec1a", FDR = 0.05, species="Mm", geneid="EntrezID")
GO.rec2 <- goana(dd.fit3, coef="com.rec2", FDR = 0.05, species="Mm", geneid="EntrezID")
GO.rec2a <- goana(dd.fit3, coef="com.rec2a", FDR = 0.05, species="Mm", geneid="EntrezID")
GO.rec3 <- goana(dd.fit3, coef="com.rec3", FDR = 0.05, species="Mm", geneid="EntrezID")

#for submission to REVIGO
top.GO.postAB <- topGO(GO.postAB,n=100)
top.GO.rec1 <- topGO(GO.rec1,n=100)
top.GO.rec1a <- topGO(GO.rec1a,n=100)
top.GO.rec2 <- topGO(GO.rec2,n=100)
top.GO.rec2a <- topGO(GO.rec2a,n=100)
#top.GO.rec3 <- topGO(GO.rec3,n=0) #no DEGs

#top 100 GO terms from each contrast submitted to Revigo using the mus musculus and 0.4 (note: there were no DEGs for rec3)
```

# Differential analysis for individual donor groups

Donor A

```
donor.day.f <- factor(interaction(AB_variables$Donor, AB_variables$Day)) #subset by donor
lev.donor.day <- c("A.0", "B.0", "A.8", "B.8", "A.11", "B.11", "A.18", "B.18")
ddf <- factor(donor.day.f, levels=lev.donor.day)

donor.day.design.mat <- model.matrix(~0+ddf)
colnames(donor.day.design.mat) <- lev.donor.day

rownames(AB_variables) == colnames(yfilt.annot)
```

```
##  [1] TRUE TRUE TRUE TRUE TRUE TRUE TRUE TRUE TRUE TRUE TRUE TRUE TRUE TRUE
## [15] TRUE TRUE TRUE TRUE TRUE TRUE TRUE TRUE TRUE TRUE TRUE TRUE TRUE TRUE
## [29] TRUE TRUE
```

```
cont.dd.mat <- makeContrasts(
  A.com.postAB = "A.8-A.0",
  B.com.postAB = "B.8-B.0",
  A.com.rec1 = "A.11-A.8",
  B.com.rec1 = "B.11-B.8",
  A.com.rec1a = "A.11-A.0",
  B.com.rec1a = "B.11-B.0",
  A.com.rec2 = "A.18-A.8",
  B.com.rec2 = "B.18-B.8",
  A.com.rec2a = "A.18-A.0",
  B.com.rec2a = "B.18-B.0",
  A.com.rec3 = "A.18-A.11",
  B.com.rec3 = "B.18-B.11",
  levels=donor.day.design.mat)
dd.fit <- lmFit(yfilt.annot, donor.day.design.mat)
dd.fit2 <- contrasts.fit(dd.fit, cont.dd.mat)
dd.fit3 <- eBayes(dd.fit2)
dd.fit3$genes$EntrezID <- rownames(dd.fit3)

A.deg.postAB <- topTable(dd.fit3, coef="A.com.postAB", adjust="BH", p.value = 0.05, number=Inf)
A.deg.rec1 <- topTable(dd.fit3, coef="A.com.rec1", adjust="BH", p.value = 0.05, number=Inf)
A.deg.rec1a <- topTable(dd.fit3, coef="A.com.rec1a", adjust="BH", p.value = 0.05, number=Inf)
A.deg.rec2 <- topTable(dd.fit3, coef="A.com.rec2", adjust="BH", p.value = 0.05, number=Inf)
A.deg.rec2a <- topTable(dd.fit3, coef="A.com.rec2a", adjust="BH", p.value = 0.05, number=Inf)
A.deg.rec3 <- topTable(dd.fit3, coef="A.com.rec3", adjust="BH", p.value = 0.05, number=Inf)
B.deg.postAB <- topTable(dd.fit3, coef="B.com.postAB", adjust="BH", p.value = 0.05, number=Inf)
B.deg.rec1 <- topTable(dd.fit3, coef="B.com.rec1", adjust="BH", p.value = 0.05, number=Inf)
B.deg.rec1a <- topTable(dd.fit3, coef="B.com.rec1a", adjust="BH", p.value = 0.05, number=Inf)
B.deg.rec2 <- topTable(dd.fit3, coef="B.com.rec2", adjust="BH", p.value = 0.05, number=Inf)
B.deg.rec2a <- topTable(dd.fit3, coef="B.com.rec2a", adjust="BH", p.value = 0.05, number=Inf)
B.deg.rec3 <- topTable(dd.fit3, coef="B.com.rec3", adjust="BH", p.value = 0.05, number=Inf)
dim(A.deg.postAB)[1]
```

```
## [1] 3
```

```
dim(A.deg.rec1)[1]
```

```
## [1] 25
```

```
dim(A.deg.rec1a)[1]
```

```
## [1] 116
```

```
dim(A.deg.rec2)[1]
```

```
## [1] 87
```

```
dim(A.deg.rec2a)[1]
```

```
## [1] 264
```

```
dim(A.deg.rec3)[1]
```

```
## [1] 0
```

```
dim(B.deg.postAB)[1]
```

```
## [1] 0
```

```
dim(B.deg.rec1)[1]
```

```
## [1] 27
```

```
dim(B.deg.rec1a)[1]
```

```
## [1] 1
```

```
dim(B.deg.rec2)[1]
```

```
## [1] 23
```

```
dim(B.deg.rec2a)[1]
```

```
## [1] 3
```

```
dim(B.deg.rec3)[1]
```

```
## [1] 0
```

```
A.GO.postAB <- goana(dd.fit3, coef="A.com.postAB", FDR = 0.05, species="Mm", geneid="EntrezID")
A.GO.rec1 <- goana(dd.fit3, coef="A.com.rec1", FDR = 0.05, species="Mm", geneid="EntrezID")
A.GO.rec1a <- goana(dd.fit3, coef="A.com.rec1a", FDR = 0.05, species="Mm", geneid="EntrezID")
A.GO.rec2 <- goana(dd.fit3, coef="A.com.rec2", FDR = 0.05, species="Mm", geneid="EntrezID")
A.GO.rec2a <- goana(dd.fit3, coef="A.com.rec2a", FDR = 0.05, species="Mm", geneid="EntrezID")
A.GO.rec3 <- goana(dd.fit3, coef="A.com.rec3", aFDR = 0.05, species="Mm", geneid="EntrezID")

#for submission to REVIGO
##when performed on 01/04/2019##

#top.A.GO.postAB <- topGO(A.GO.postAB,n=0) #only 3 DEGs so not included
top.A.GO.rec1 <- topGO(A.GO.rec1,n=100)[topGO(A.GO.rec1,n=100)[,4] + topGO(A.GO.rec1,n=100)[,5] > 3,] #57 GO terms retained when performed on 01/04/2019
top.A.GO.rec1a <- topGO(A.GO.rec1a,n=100)[topGO(A.GO.rec1a,n=100)[,4] + topGO(A.GO.rec1a,n=100)[,5] > 3,] #18 GO terms retained when performed on 01/04/2019
top.A.GO.rec2 <- topGO(A.GO.rec2,n=100)[topGO(A.GO.rec2,n=100)[,4] + topGO(A.GO.rec2,n=100)[,5] > 3,] #73 GO terms retained when performed on 01/04/2019
top.A.GO.rec2a <- topGO(A.GO.rec2a,n=100)[topGO(A.GO.rec2a,n=100)[,4] + topGO(A.GO.rec2a,n=100)[,5] > 3,] #84 GO terms retained when performed on 01/04/2019
#top.A.GO.rec3 <- topGO(A.GO.rec3,n=0) #0 DEGs so not included

B.GO.postAB <- goana(dd.fit3, coef="B.com.postAB", FDR = 0.05, species="Mm", geneid="EntrezID")
B.GO.rec1 <- goana(dd.fit3, coef="B.com.rec1", FDR = 0.05, species="Mm", geneid="EntrezID")
B.GO.rec1a <- goana(dd.fit3, coef="B.com.rec1a", FDR = 0.05, species="Mm", geneid="EntrezID")
B.GO.rec2 <- goana(dd.fit3, coef="B.com.rec2", FDR = 0.05, species="Mm", geneid="EntrezID")
B.GO.rec2a <- goana(dd.fit3, coef="B.com.rec2a", FDR = 0.05, species="Mm", geneid="EntrezID")
B.GO.rec3 <- goana(dd.fit3, coef="B.com.rec3", FDR = 0.05, species="Mm", geneid="EntrezID")

#for submission to REVIGO
##performed on 01/04/2019##

#top.B.GO.postAB <- topGO(B.GO.postAB,n=0) #no DEGs so not included
top.B.GO.rec1 <- topGO(B.GO.rec1,n=100)[topGO(B.GO.rec1,n=100)[,4] + topGO(B.GO.rec1,n=100)[,5] > 3,] #52 GO terms when performed on 01/04/2019
#top.B.GO.rec1a <- topGO(B.GO.rec1a,n=20) #only 1 DEG so not included
top.B.GO.rec2 <- topGO(B.GO.rec2,n=20)[topGO(B.GO.rec2,n=100)[,4] + topGO(B.GO.rec2,n=100)[,5] > 3,] #67 GO terms when performed on 01/04/2019
#top.B.GO.rec2a <- topGO(B.GO.rec2a,n=20) #only 3 DEGs so not included
#top.B.GO.rec3 <- topGO(B.GO.rec3,n=20) #no DEGs so not included


#only pathways with >3 contributing genes were selected. All resulting pathways included for submission to REVIGO for both donor groups had p-values <0.01.
```

# Plotting Revigo output

This part requires manual assembly of the Revigo output which is not presented here

```
#REVIGO - these values have been pre-calculated and formatted for plotting
Revigo.combined.all <- read.table("Revigo_output_combined_analysis.txt", header = T, sep = "\t")

Revigo.combined.all$description <- as.character(Revigo.combined.all$description)
Revigo.combined.all[grepl("transmembrane rec", Revigo.combined.all$description),2] <- "transmembrane receptor protein Ser/Thr kinase signaling path"
my_palette <- c("pink", "lightgreen", "lightblue")
Revigo.combined.all$Contrast <- factor(Revigo.combined.all$Contrast, levels = c("D8vD0", "D11vD8", "D11vD0", "D18vD8", "D18vD0"))

start.vec <- seq(1, nrow(Revigo.combined.all), 1)
ord.vec <- c()

for ( i in 1:nrow(Revigo.combined.all)){
  if (isUnique(Revigo.combined.all$description)[i] == FALSE & Revigo.combined.all$description[i] %in% Revigo.combined.all$description[1:i-1] == FALSE){
    x <- start.vec[Revigo.combined.all$description == Revigo.combined.all$description[i]]
    ord.vec <- c(ord.vec, x)
  } else if (isUnique(Revigo.combined.all$description)[i] == FALSE & Revigo.combined.all$description[i] %in% Revigo.combined.all$description[1:i-1] == TRUE){
    ord.vec <- ord.vec
  } else {
    y <- start.vec[Revigo.combined.all$description == Revigo.combined.all$description[i]]
    ord.vec <- c(ord.vec, y)
  }
}
Revigo.combined.all <- Revigo.combined.all[ord.vec,]
Revigo.combined.all.filt <- Revigo.combined.all[abs(Revigo.combined.all$log10_p_value) >= 5, ]

p.combined <- ggplot(Revigo.combined.all.filt, aes(x=description, y=-log10_p_value, fill = Domain)) + geom_bar(stat="identity", width = 0.6) + facet_grid(~Contrast, scales="fixed") + scale_x_discrete("",limits = unique(c(rev(as.character(Revigo.combined.all.filt$description))))) + coord_flip() + ylab("-log10 (p Value)") + theme_classic() + scale_fill_manual(values = my_palette, labels = c("Biological Process", "Cellular Component", "Molecular Function"))

#Donor A

Revigo.A.all <- read.table("Revigo_output_donor_A.txt", header = T, sep = "\t")

start.vec <- seq(1, nrow(Revigo.A.all), 1)
ord.vec <- c()

for ( i in 1:nrow(Revigo.A.all)){
  if (isUnique(Revigo.A.all$description)[i] == FALSE & Revigo.A.all$description[i] %in% Revigo.A.all$description[1:i-1] == FALSE){
    x <- start.vec[Revigo.A.all$description == Revigo.A.all$description[i]]
    ord.vec <- c(ord.vec, x)
  } else if (isUnique(Revigo.A.all$description)[i] == FALSE & Revigo.A.all$description[i] %in% Revigo.A.all$description[1:i-1] == TRUE){
    ord.vec <- ord.vec
  } else {
    y <- start.vec[Revigo.A.all$description == Revigo.A.all$description[i]]
    ord.vec <- c(ord.vec, y)
  }
}
Revigo.A.all <- Revigo.A.all[ord.vec,]

p.A <- ggplot(Revigo.A.all, aes(x=description, y=-log10_p_value, fill = Domain)) + geom_bar(stat="identity", width = 0.6) + facet_grid(~Contrast, scales="fixed") + scale_x_discrete("",limits = unique(c(rev(as.character(Revigo.A.all$description))))) + coord_flip() + ylab("-log10 (p Value)") + theme_classic() + scale_fill_manual(values = my_palette, labels = c("Biological Process", "Cellular Component", "Molecular Function"))

Revigo.B.all <- read.table("Revigo_output_donor_B.txt", header = T, sep = "\t")

start.vec <- seq(1, nrow(Revigo.B.all), 1)
ord.vec <- c()

for ( i in 1:nrow(Revigo.B.all)){
  if (isUnique(Revigo.B.all$description)[i] == FALSE & Revigo.B.all$description[i] %in% Revigo.B.all$description[1:i-1] == FALSE){
    x <- start.vec[Revigo.B.all$description == Revigo.B.all$description[i]]
    ord.vec <- c(ord.vec, x)
  } else if (isUnique(Revigo.B.all$description)[i] == FALSE & Revigo.B.all$description[i] %in% Revigo.B.all$description[1:i-1] == TRUE){
    ord.vec <- ord.vec
  } else {
    y <- start.vec[Revigo.B.all$description == Revigo.B.all$description[i]]
    ord.vec <- c(ord.vec, y)
  }
}
Revigo.B.all <- Revigo.B.all[ord.vec,]

p.B <- ggplot(Revigo.B.all, aes(x=description, y=-log10_p_value, fill = Domain)) + geom_bar(stat="identity", width = 0.6) + facet_grid(~Contrast, scales="fixed") + scale_x_discrete("",limits = unique(c(rev(as.character(Revigo.B.all$description))))) + coord_flip() + ylab("-log10 (p Value)") + theme_classic() + scale_fill_manual(values = my_palette, labels = c("Biological Process", "Molecular Function"))
```

# Plot GO terms retained by REVIGO

```
p.combined
```

```
p.A
```

```
p.B
```

Donor B

# Heatmaps

```
library(gplots)

#DEGs
#combined data contrast Day 18 vs Day 0
yfilt.annot.rec2a <- yfilt.annot[rownames(yfilt.annot) %in% rownames(deg.rec2a),]
deg.rec2a <- deg.rec2a[order(deg.rec2a$logFC),]
yfilt.annot.rec2a <- yfilt.annot.rec2a[match(rownames(deg.rec2a), rownames(yfilt.annot.rec2a)),]

colourmap <- function (Day) { if (Day=="0") "#66C2A5" else if (Day=="8") "#FC8D62" else if (Day=="11") "#8DA0CB" else "#E78AC3"}
patientcolors <- unlist(lapply(AB_variables$Day, colourmap))
```

# Day 18 vs Day 0

Combined DEGs

```
heatmap.2(as.matrix(yfilt.annot.rec2a),key=T,trace='none',scale="row", hclustfun = function(x) hclust(x,method = 'ward.D'), density.info="none",dendrogram="col",Rowv = FALSE,Colv=TRUE, cexRow = 1.3,cexCol = 1,col=greenred(256), margins=c(5,20), symm=F,symkey=F,symbreaks=T, ColSideColors = patientcolors, keysize = 1.75)
```

```
#DEGs when analysed individually
#Day 11 vs Day 8
unique.A.rec1 <- rownames(A.deg.rec1[!(rownames(A.deg.rec1) %in% rownames(B.deg.rec1)),])
shared.rec1 <- intersect(rownames(A.deg.rec1), rownames(B.deg.rec1))
unique.B.rec1 <- rownames(B.deg.rec1[!(rownames(B.deg.rec1) %in% rownames(A.deg.rec1)),])
deg.names.rec1 <- c(unique.A.rec1, unique.B.rec1, shared.rec1)
deg.names.rec1 <- cbind(Genes = deg.names.rec1, Profile = c(rep("A", length(unique.A.rec1)), rep("B", length(unique.B.rec1)), rep("S", length(shared.rec1))), Colour.contrasts = c(rep("#FF0000", length(unique.A.rec1)), rep("#000080", length(unique.B.rec1)), rep("#838B8B", length(shared.rec1))))
order.rec1 <- rbind(A.deg.rec1, B.deg.rec1[rownames(B.deg.rec1) %in% unique.B.rec1,])
order.rec1 <- order.rec1[order(order.rec1$logFC),]
yfilt.rec1 <- yfilt.annot[rownames(yfilt.annot) %in% deg.names.rec1[,1],]
yfilt.rec1 <- yfilt.rec1[match(rownames(order.rec1), rownames(yfilt.rec1)),]
deg.names.rec1 <- deg.names.rec1[match(rownames(order.rec1), deg.names.rec1),]
```

# Day 11 vs Day 8

Individual DEGs

```
heatmap.2(as.matrix(yfilt.rec1),key=T,trace='none',scale="row", hclustfun = function(x) hclust(x,method = 'ward.D'), density.info="none",dendrogram="col",Rowv = FALSE,Colv=TRUE, cexRow = 1.3,cexCol = 1,col=greenred(256), margins=c(5,20), symm=F,symkey=F,symbreaks=T, ColSideColors = patientcolors, keysize = 1.75, RowSideColors = deg.names.rec1[,3])
```

```
#Day 18 vs Day 8
unique.A.rec2 <- rownames(A.deg.rec2[!(rownames(A.deg.rec2) %in% rownames(B.deg.rec2)),])
shared.rec2 <- intersect(rownames(A.deg.rec2), rownames(B.deg.rec2))
unique.B.rec2 <- rownames(B.deg.rec2[!(rownames(B.deg.rec2) %in% rownames(A.deg.rec2)),])
deg.names.rec2 <- c(unique.A.rec2, unique.B.rec2, shared.rec2)
deg.names.rec2 <- cbind(Genes = deg.names.rec2, Profile = c(rep("A", length(unique.A.rec2)), rep("B", length(unique.B.rec2)), rep("S", length(shared.rec2))), Colour.contrasts = c(rep("#FF0000", length(unique.A.rec2)), rep("#000080", length(unique.B.rec2)), rep("#838B8B", length(shared.rec2))))
order.rec2 <- rbind(A.deg.rec2, B.deg.rec2[rownames(B.deg.rec2) %in% unique.B.rec2,])
order.rec2 <- order.rec2[order(order.rec2$logFC),]
yfilt.rec2 <- yfilt.annot[rownames(yfilt.annot) %in% deg.names.rec2[,1],]
yfilt.rec2 <- yfilt.rec2[match(rownames(order.rec2), rownames(yfilt.rec2)),]
deg.names.rec2 <- deg.names.rec2[match(rownames(order.rec2), deg.names.rec2),]
```

# Day 18 vs Day 8

Individual DEGs

```
heatmap.2(as.matrix(yfilt.rec2),key=T,trace='none',scale="row", hclustfun = function(x) hclust(x,method = 'ward.D'), density.info="none",dendrogram="col",Rowv = FALSE,Colv=TRUE, cexRow = 1.3,cexCol = 1,col=greenred(256), margins=c(5,20), symm=F,symkey=F,symbreaks=T, ColSideColors = patientcolors, keysize = 1.75, RowSideColors = deg.names.rec2[,3])
```

## Venn diagrams

These do not project well - included for code

```
#library(VennDiagram)

#Day 11 vs Day 8
#draw.pairwise.venn(area1 = nrow(A.deg.rec1), area2 = nrow(B.deg.rec1),  cross.area = length(intersect(rownames(A.deg.rec1), rownames(B.deg.rec1))), col = c("red", "blue"), scaled = T, inverted = T, fill = c("red", "blue"), cex = 6, label.col = c("black", "white", "black"), fontfamily = "sans")

#Day 11 vs Day 0
#draw.pairwise.venn(area1 = nrow(A.deg.rec1a), area2 = nrow(B.deg.rec1a),  cross.area = length(intersect(rownames(A.deg.rec1a), rownames(B.deg.rec1a))), col = c("red", "blue"), scaled = T, fill = c("red", "blue"), cex = 6, label.col = c("black", "white", "black"), fontfamily = "sans")

#Day 18 vs Day 8
#draw.pairwise.venn(area1 = nrow(A.deg.rec2), area2 = nrow(B.deg.rec2),  cross.area = length(intersect(rownames(A.deg.rec2), rownames(B.deg.rec2))), col = c("red", "blue"), scaled = T, fill = c("red", "blue"), cex = 6, label.col = c("black", "white", "black"), fontfamily = "sans")

#Day 18 vs Day 0
#draw.pairwise.venn(area1 = nrow(A.deg.rec2a), area2 = nrow(B.deg.rec2a),  cross.area = length(intersect(rownames(A.deg.rec2a), rownames(B.deg.rec2a))), col = c("red", "blue"), scaled = T, fill = c("red", "blue"), cex = 6, label.col = c("black", "black", "black"), fontfamily = "sans")
```

# Circadian rhythm genes

```
#supplementary figure 3 heatmap
circadian_genes <- c("Cipc", "Tef", "Dbp", "Noct", "Prkaa2", "Per1", "Usp2", "Ghrl", "Nr1d2", "Arntl", "Nfil3", "Per2", "Kdm5b", "Cry1", "Npas2", "Ciart", "Per3")
all(rownames(yfilt.annot) == entrezid$NCBI.gene.ID)
```

```
## [1] TRUE
```

```
yfilt.sym <- yfilt.annot
rownames(yfilt.sym) <- entrezid$MGI.symbol
yfilt.circ <- yfilt.sym[rownames(yfilt.sym) %in% circadian_genes,]

heatmap.2(as.matrix(yfilt.circ),key=T,trace='none',scale="row", density.info="none",dendrogram="none",Rowv = TRUE,Colv=FALSE, cexRow = 1.3,cexCol = 1,col=greenred(256), margins=c(5,20), symm=F,symkey=F,symbreaks=T, ColSideColors = patientcolors, keysize = 1.75)
```

# GSVA, HALLA

```
###### HAllA correlation

# Step 1: create individual OTU matrices (genus) for each donor group
library(phyloseq)
library(dplyr)

ps2 <- readRDS("ps_final_filtered_microbiota.RData")
ps.genus <- tax_glom(ps2, "Genus")
ps.genus <- transform_sample_counts(ps.genus, function(x) x/sum(x))
otu.genus <- otu_table(ps.genus)
all(colnames(otu.genus) == rownames(tax_table(ps.genus)))
```

```
## [1] TRUE
```

```
colnames(otu.genus) <- tax_table(ps.genus)[,6]
rownames(otu.genus) <- gsub("co", "D", rownames(otu.genus))

# Step 2: rearrange AB_variables to subset OTU tables for donors
AB_variables_reordered_corr <- AB_variables[match(rownames(otu.genus), rownames(AB_variables)),]

# Step 3: subset OTU tables and remove rows with 0 abundance

otu.genus.A <- otu.genus[AB_variables_reordered_corr$Donor == "A",]
otu.genus.B <- otu.genus[AB_variables_reordered_corr$Donor == "B",]
otu.genus.A <- otu.genus.A[,!(colSums(otu.genus.A) == 0)]
otu.genus.B <- otu.genus.B[,!(colSums(otu.genus.B) == 0)]

# Step 4: Get GO pathway expression for combined donors from GSVA script
#     http://bioinf.wehi.edu.au/software/MSigDB/ for MSigDB for mice


load("mouse_c5_v5p2.rdata")

phenoData <- new("AnnotatedDataFrame", data=as.data.frame(AB_variables), varMetadata=as.data.frame(colnames(AB_variables)))
eSet <- ExpressionSet(assayData=as.matrix(yfilt.annot), phenoData=phenoData, annotation = "org.Mm.eg.db")
fData(eSet)$PROBEID <- as.character(rownames(fData(eSet)))
entrezid <- cbind(entrezid[,1:5], PROBEID = as.character(entrezid$NCBI.gene.ID))
fData(eSet) <- left_join(fData(eSet), entrezid)


### Combined
filtered_eset <- nsFilter(eSet, require.entrez=TRUE, remove.dupEntrez=TRUE, var.func=IQR, var.filter=TRUE, var.cutoff=0.5, filterByQuantile=TRUE)

AB_eset_filt <- filtered_eset$eset
AB_es <- gsva(AB_eset_filt, Mm.c5, min.sz=10, max.sz=9999, parallel.sz = 2, abs.ranking=FALSE, verbose=TRUE)
```

```
## Estimating GSVA scores for 5308 gene sets.
## Computing observed enrichment scores
## Estimating ECDFs with Gaussian kernels
## Allocating cluster
## Estimating enrichment scores in parallel
## Taking diff of max KS.
## Cleaning up
```

```
donor.day.f <- factor(paste("d", AB_es$Day, sep = ".")) #subset by donor
lev.donor.day <- c("d.0", "d.8", "d.11", "d.18")
ddf <- factor(donor.day.f, levels=lev.donor.day)
donor.day.design.mat <- model.matrix(~0+ddf)
colnames(donor.day.design.mat) <- c(lev.donor.day)
cont.dd.mat <- makeContrasts(
  com.postAB = "d.8-d.0",
  com.rec1 = "d.11-d.8",
  com.rec1a = "d.11-d.0",
  com.rec2 = "d.18-d.8",
  com.rec2a = "d.18-d.0",
  com.rec3 = "d.18-d.11",
  levels=donor.day.design.mat)
dd.fit <- lmFit(AB_es, donor.day.design.mat)
dd.fit2 <- contrasts.fit(dd.fit, cont.dd.mat)
dd.fit3 <- eBayes(dd.fit2)
dd.fit3$genes$EntrezID <- rownames(dd.fit3)

deg.postAB <- topTable(dd.fit3, coef="com.postAB", adjust="BH", p.value = 0.01, number=Inf)
deg.rec1 <- topTable(dd.fit3, coef="com.rec1", adjust="BH", p.value = 0.01, number=Inf)
deg.rec1a <- topTable(dd.fit3, coef="com.rec1a", adjust="BH", p.value = 0.01, number=Inf)
deg.rec2 <- topTable(dd.fit3, coef="com.rec2", adjust="BH", p.value = 0.01, number=Inf)
deg.rec2a <- topTable(dd.fit3, coef="com.rec2a", adjust="BH", p.value = 0.01, number=Inf)
deg.rec3 <- topTable(dd.fit3, coef="com.rec3", adjust="BH", p.value = 0.01, number=Inf)
dim(deg.postAB)[1]
```

```
## [1] 0
```

```
dim(deg.rec1)[1]
```

```
## [1] 323
```

```
dim(deg.rec1a)[1]
```

```
## [1] 120
```

```
dim(deg.rec2)[1]
```

```
## [1] 171
```

```
dim(deg.rec2a)[1]
```

```
## [1] 100
```

```
dim(deg.rec3)[1]
```

```
## [1] 0
```

```
# concatenate all together in single object
GO_enrich_all <- rbind(cbind(deg.postAB, Contrast = rep("D8vsD0", nrow(deg.postAB))), cbind(deg.rec1, Contrast = rep("D11vsD8", nrow(deg.rec1))), cbind(deg.rec1a, Contrast = rep("D11vsD0", nrow(deg.rec1a))), cbind(deg.rec2, Contrast = rep("D18vsD8", nrow(deg.rec2))), cbind(deg.rec2a, Contrast = rep("D18vsD0", nrow(deg.rec2a))), cbind(deg.rec3, Contrast = rep("D18vsD11", nrow(deg.rec3))))

GO_enrich_all$Contrast <- as.character(GO_enrich_all$Contrast)

GO_enrich_all$Contrast <- factor(GO_enrich_all$Contrast, levels = c("D8vsD0", "D11vsD0", "D11vsD8", "D18vsD0", "D18vsD8", "D18vsD11"))


GO_enrich_all <- GO_enrich_all[abs(GO_enrich_all$logFC) >= 0.5,]
uniquePathwayIDs <- rev(unique(rev(as.character(GO_enrich_all$EntrezID))))

heat <- as.matrix(exprs(AB_es[uniquePathwayIDs,]))
heat <- as.matrix(exprs(AB_es[uniquePathwayIDs,]))
heat <- heat[,match(rownames(AB_variables_reordered_corr), colnames(heat))]

heat.com.A <- heat[,AB_variables_reordered_corr$Donor == "A"]
heat.com.B <- heat[,AB_variables_reordered_corr$Donor == "B"]

# write tables for HAllA, ensuring matching sample names!

colnames(t(otu.genus.A)) == colnames(heat.com.A)
```

```
##  [1] TRUE TRUE TRUE TRUE TRUE TRUE TRUE TRUE TRUE TRUE TRUE TRUE TRUE TRUE
## [15] TRUE
```

```
colnames(t(otu.genus.B)) == colnames(heat.com.B)
```

```
##  [1] TRUE TRUE TRUE TRUE TRUE TRUE TRUE TRUE TRUE TRUE TRUE TRUE TRUE TRUE
## [15] TRUE
```

```
write.table(heat.com.A, "heat.com.A.txt", sep = "\t", quote = F)
write.table(heat.com.B, "heat.com.B.txt", sep = "\t", quote = F)
write.table(t(otu.genus.A), "otu.genus.A.txt", sep = "\t", quote = F)
write.table(t(otu.genus.B), "otu.genus.B.txt", sep = "\t", quote = F)


# use as input for HAllA

#http://huttenhower.sph.harvard.edu/halla

#commands below used. If doing this step, replace the files in the Halla_files directory (similarity_table.txt and p_values.txt) with your own output from Halla in the next step
#note that the output files from above need to have a '#' added at the very first position (row 1, column 1) followed by a tab space to be acceptably formatted for Halla
#note that output files from Halla will need to have this '#' removed for graphing below
#halla v 0.8.17
#halla -X otu.genus.A.txt -Y heat.com.A.txt -m spearman --output donorA_halla -q 0.1
#halla -X otu.genus.B.txt -Y heat.com.B.txt -m spearman --output donorB_halla -q 0.1
```

# Network plot

Read in Halla output (or use your own as described above)

```
###### Graph Halla output

library(corrr)
library(igraph)
library(ggraph)
library(tidyr)
library(dplyr)

A.com.halla.r <- read.table("Halla_files/similarity_table.txt", header = T)
A.com.halla.p <- read.table("Halla_files/pvalues_table.txt", header = T)
layout_pre <- read.table("layout_pre.txt", header = T, sep = "\t")


A.com.halla.r <- cbind(Genera = rownames(A.com.halla.r), A.com.halla.r)
A.com.halla.p <- cbind(Genera = rownames(A.com.halla.p), A.com.halla.p)

A.com.halla.r.long <- gather(A.com.halla.r, key = Pathway, value = Correlation, GO_TRANSFERASE_ACTIVITY_TRANSFERRING_NITROGENOUS_GROUPS:GO_DNA_DEALKYLATION)
A.com.halla.p.long <- gather(A.com.halla.p, key = Pathway, value = p.value, GO_TRANSFERASE_ACTIVITY_TRANSFERRING_NITROGENOUS_GROUPS:GO_DNA_DEALKYLATION)
A.com.halla.p.long$p.value <- p.adjust(A.com.halla.p.long$p.value, "fdr")

A.com.halla.r.long <- A.com.halla.r.long[A.com.halla.p.long$p.value < 0.1,]

graph_cors <- A.com.halla.r.long %>%
  graph_from_data_frame(directed = FALSE)

graph_cors_genus <- A.com.halla.r.long

V(graph_cors)$Class <- c(rep("Genera", length(unique(graph_cors_genus$Genera))), rep("Pathway", length(unique(graph_cors_genus$Pathway))))
genera_after_filtering <- as.character(unique(graph_cors_genus$Genera))
modules_after_filtering <- as.character(unique(graph_cors_genus$Pathway))
V(graph_cors)$lab.genus <- c(genera_after_filtering, rep("", length(unique(graph_cors_genus$Pathway))))
V(graph_cors)$lab.pathways2 <- as.character(layout_pre$lab.pathways2)
V(graph_cors)$lab.colours <- as.character(layout_pre$lab.pathways3)


#to replicate exactly, follow steps (manual moving of points in 'layout' df)
set.seed(40) #for both
layout <- create_layout(graph_cors, layout = 'nicely')
layout_pre <- layout_pre[match(layout$name, layout_pre$name),]
col.vec <- c()

for(i in 1:length(levels(layout$lab.pathways2))){
  col.x <- unique(as.character(layout[layout$lab.pathways2 == levels(layout$lab.pathways2)[i],]$lab.colours))
  col.vec <- c(col.vec, col.x)
}

layout$lab.colours <- factor(layout$lab.colours, levels = col.vec)

p.numeric.modules <- ggraph(layout) +
  geom_edge_link2(aes(color = Correlation)) +
  guides(edge_alpha = "none", edge_width = "none") +
  scale_edge_colour_gradient2("Correlation", low = "blue", mid = "white", high = "red") +
  theme_graph() + scale_color_manual("GO terms", values = as.character(levels(layout$lab.colours))) +
  labs(title = "Donor A") +
  scale_shape_manual(values=c(16, 15)) + geom_node_point(aes(shape = Class, color = as.character(layout$lab.pathways2)), size = 4.5) + geom_node_label(aes(label=lab.genus), repel = T)
```

# Plot network plot

```
p.numeric.modules
```

# Session info

```
sessionInfo()
```

```
## R version 3.6.0 (2019-04-26)
## Platform: x86_64-pc-linux-gnu (64-bit)
## Running under: Ubuntu 18.04.2 LTS
## 
## Matrix products: default
## BLAS:   /usr/lib/x86_64-linux-gnu/blas/libblas.so.3.7.1
## LAPACK: /usr/lib/x86_64-linux-gnu/lapack/liblapack.so.3.7.1
## 
## locale:
##  [1] LC_CTYPE=en_US.UTF-8       LC_NUMERIC=C              
##  [3] LC_TIME=fr_FR.UTF-8        LC_COLLATE=en_US.UTF-8    
##  [5] LC_MONETARY=fr_FR.UTF-8    LC_MESSAGES=en_US.UTF-8   
##  [7] LC_PAPER=fr_FR.UTF-8       LC_NAME=C                 
##  [9] LC_ADDRESS=C               LC_TELEPHONE=C            
## [11] LC_MEASUREMENT=fr_FR.UTF-8 LC_IDENTIFICATION=C       
## 
## attached base packages:
## [1] parallel  stats4    stats     graphics  grDevices utils     datasets 
## [8] methods   base     
## 
## other attached packages:
##  [1] tidyr_0.8.3           ggraph_1.0.2          igraph_1.2.4.1       
##  [4] corrr_0.3.2           dplyr_0.8.0.1         phyloseq_1.24.2      
##  [7] gplots_3.0.1.1        GO.db_3.8.2           factoextra_1.0.5     
## [10] ade4_1.7-13           ggplot2_3.1.1         preprocessCore_1.46.0
## [13] snow_0.4-3            genefilter_1.62.0     GSVA_1.32.0          
## [16] GSEABase_1.46.0       graph_1.62.0          annotate_1.58.0      
## [19] XML_3.98-1.19         org.Mm.eg.db_3.8.2    AnnotationDbi_1.46.0 
## [22] IRanges_2.18.0        S4Vectors_0.22.0      Biobase_2.42.0       
## [25] BiocGenerics_0.30.0   statmod_1.4.30        knitr_1.22           
## [28] limma_3.36.5         
## 
## loaded via a namespace (and not attached):
##  [1] colorspace_1.4-1   XVector_0.24.0     ggpubr_0.2        
##  [4] farver_1.1.0       ggrepel_0.8.1      bit64_0.9-7       
##  [7] codetools_0.2-16   splines_3.6.0      geneplotter_1.58.0
## [10] shinythemes_1.1.2  polyclip_1.10-0    jsonlite_1.6      
## [13] cluster_2.0.8      ggforce_0.2.2      shiny_1.3.2       
## [16] compiler_3.6.0     assertthat_0.2.1   Matrix_1.2-17     
## [19] lazyeval_0.2.2     later_0.8.0        tweenr_1.0.1      
## [22] htmltools_0.3.6    tools_3.6.0        gtable_0.3.0      
## [25] glue_1.3.1         reshape2_1.4.3     Rcpp_1.0.1        
## [28] Biostrings_2.52.0  multtest_2.36.0    gdata_2.18.0      
## [31] ape_5.3            nlme_3.1-139       iterators_1.0.10  
## [34] xfun_0.6           stringr_1.4.0      mime_0.6          
## [37] gtools_3.8.1       zlibbioc_1.28.0    MASS_7.3-51.1     
## [40] scales_1.0.0       promises_1.0.1     biomformat_1.12.0 
## [43] rhdf5_2.24.0       RColorBrewer_1.1-2 yaml_2.2.0        
## [46] memoise_1.1.0      gridExtra_2.3      stringi_1.4.3     
## [49] RSQLite_2.1.1      foreach_1.4.4      permute_0.9-5     
## [52] caTools_1.17.1.2   rlang_0.3.4        pkgconfig_2.0.2   
## [55] bitops_1.0-6       evaluate_0.13      lattice_0.20-38   
## [58] purrr_0.3.2        Rhdf5lib_1.2.1     labeling_0.3      
## [61] bit_1.1-14         tidyselect_0.2.5   plyr_1.8.4        
## [64] magrittr_1.5       R6_2.4.0           DBI_1.0.0         
## [67] pillar_1.4.0       withr_2.1.2        mgcv_1.8-28       
## [70] survival_2.43-3    RCurl_1.95-4.12    tibble_2.1.1      
## [73] crayon_1.3.4       KernSmooth_2.23-15 rmarkdown_1.12    
## [76] viridis_0.5.1      grid_3.6.0         data.table_1.12.2 
## [79] blob_1.1.1         vegan_2.5-5        digest_0.6.18     
## [82] xtable_1.8-4       httpuv_1.5.1       munsell_0.5.0     
## [85] viridisLite_0.3.0
```
